# RhoA-mediated G_12_-G_13_ signaling maintains muscle stem cell quiescence and prevents stem cell loss

**DOI:** 10.1038/s41421-024-00696-7

**Published:** 2024-07-16

**Authors:** Yundong Peng, Jingjing Du, Rui Li, Stefan Günther, Nina Wettschureck, Stefan Offermanns, Yan Wang, Andre Schneider, Thomas Braun

**Affiliations:** 1 Department of Cardiac Development and Remodeling, Max Planck Institute for Heart and Lung Research, Bad Nauheim, Germany; 2 Department of Pharmacology, Max Planck Institute for Heart and Lung Research, Bad Nauheim, Germany; 3 Member of the German Center for Cardiovascular Research (DZHK), member of the German Center for Lung Research (DZL), Berlin, Germany; 4 College of Animal Science and Technology, Sichuan Agricultural University, Chengdu, Sichuan, China

**Keywords:** Muscle stem cells, Quiescence

## Abstract

Multiple processes control quiescence of muscle stem cells (MuSCs), which is instrumental to guarantee long-term replenishment of the stem cell pool. Here, we describe that the G-proteins G_12_-G_13_ integrate signals from different G-protein-coupled receptors (GPCRs) to control MuSC quiescence via activation of RhoA. Comprehensive screening of GPCR ligands identified two MuSC-niche-derived factors, endothelin-3 (ET-3) and neurotensin (NT), which activate G_12_-G_13_ signaling in MuSCs. Stimulation with ET-3 or NT prevented MuSC activation, whereas pharmacological inhibition of ET-3 or NT attenuated MuSC quiescence. Inactivation of *Gna12*-*Gna13* or *Rhoa* but not of *Gnaq-Gna11* completely abrogated MuSC quiescence, which depleted the MuSC pool and was associated with accelerated sarcopenia during aging. Expression of constitutively active RhoA prevented exit from quiescence in *Gna12*-*Gna13* mutant MuSCs, inhibiting cell cycle entry and differentiation via Rock and formins without affecting Rac1-dependent MuSC projections, a hallmark of quiescent MuSCs. The study uncovers a critical role of G_12_-G_13_ and RhoA signaling for active regulation of MuSC quiescence.

## Introduction

Skeletal muscle stem cells (MuSCs) are crucial for postnatal muscle growth, long-term maintenance of muscle mass, and muscle regeneration. Sustained survival of MuSCs and replenishment of the stem cell pool critically depend on the acquisition of a resting, quiescent state. Loss of MuSC quiescence during aging impairs self-renewal and reduces the number of MuSCs^1^. Compromised quiescence and self-renewal along with increased senescence of MuSCs in geriatric mice has been associated with decreased muscle mass, although direct evidence that loss of MuSC quiescence enhances sarcopenia is missing so far^2^. The situation is further complicated by findings that nearly complete ablation of MuSC does not cause sarcopenia under base line conditions^3,4^.

Quiescent MuSCs are characterized by cell cycle withdrawal, translational suppression, epigenetic inactivation of myogenic regulatory factors (e.g., *Myf5*, *MyoD*), and increased expression of *Pax7*^2,5–7^. Numerous mechanisms have been described which secure quiescence of MuSCs, ranging from diffusible extracellular cues, signals from the MuSC niche, and transcription factor circuits to epigenetic mechanisms^2^. For example, muscle fiber-derived Wnt4 suppresses MuSC activation through RhoA and increased supply of Wnt4 arrests MuSCs in a deep state of quiescence, delaying muscle repair^8^. Similarly, collagen V (COLV), secreted by MuSCs, is a surrogate ligand for the calcitonin receptor (CalcR) and constitutes an important component of the quiescent niche, as demonstrated by aberrant cell cycle entry of MuSCs after inactivation of COLV^9^. The sheer number of processes regulating quiescence is staggering, raising questions how integration of signals is achieved to control the eventual outcome. Coordination of parallel signaling processes is critical, probably requiring intracellular integration for orchestrating the regulatory network that controls the quiescence state of MuSC^10^.

Extracellular signals are often relayed into cells via G-protein-coupled receptors (GPCRs), which belong to a large superfamily of proteins with seven transmembrane domains. GPCRs regulate such diverse processes as growth, proliferation, differentiation, secretion, and metabolism. Canonical GPCR signaling is mediated by different heterotrimeric G-proteins (Gα, Gβ, and Gγ), which are activated by GPCRs, facilitating exchange of GDP by GTP on the Gα subunit upon ligand binding. In addition, unconventional GPCRs exist, such as the WNT receptors, which belong to the Class Frizzled of GPCRs. Frizzled receptors mainly signal via the WNT/β-catenin, WNT/calcium, or planar cell polarity (PCP) pathways, the latter leading to activation of RhoA and ROCK^11^. Activated GPCRs regulate different subfamilies of heterotrimeric G-proteins, including G_s_, G_i_-G_o_, G_q_-G_11_, and G_12_-G_13_, which activate different intracellular signaling pathways^12^. Members of the G_s_ and G_i_-G_o_ family antagonistically regulate adenylyl cyclases and thereby intracellular cAMP concentrations. The prototypical marker of quiescent MuSCs, CalcR, is coupled to G_s_ and signals via adenylyl cyclase and cAMP^9,13^. However, inactivation of *Calcr* has only moderate effects on MuSC quiescence^13^. Activation of G_q_-G_11_ by GPCRs primarily increases PLC-β activity, leading to PKC and Ca^2+^ signaling. G_q_-G_11_-coupled receptors do not appear to discriminate between G_q_ and G_11_ and no differences in the ability to regulate phospholipase C-isoforms were observed. The G-proteins G_12_-G_13_ are often activated by receptors that also couple to G_q_-G_11_, but initiate various signaling pathways, including RhoA-dependent processes. Genetic studies indicated a strong functional overlap between G_q_ and G_11_ as well as between G_12_ and G_13_, requiring the use of *Gnaq*-*Gna11* and *Gna12*-*Gna13* compound mutant mice for functional studies^12^.

Several GPCRs such as CalcR and the adhesion GPCR GPR116, also known as AGDRF5, which increases the nuclear localization of β-arrestin, thereby enabling interactions with cAMP response element-binding protein, are known to regulate MuSC quiescence^13,14^. However, a systematic analysis of the role of canonical GPCRs in the regulation of MuSCs is missing. Likewise, the potential function of G_q_-G_11_ and G_12_-G_13_ for integrating activities of GPCRs in MuSCs or the role of signaling pathways downstream of these G-proteins have not been analyzed so far. In a combination of hypothesis-driven and bias-free approaches, we comprehensively assessed expression of GPCRs in quiescent MuSCs and determined the function of individual GPCRs for preventing activation of MuSCs using a pharmacological screening approach. Two niche-derived GPCR ligands were identified, endothelin-3 (ET-3) and neurotensin (NT), which activate the G_12_-G_13_ pathway. Genetic inactivation of *Gnaq*-*Gna11* and *Gna12*-*Gna13* as well as expression of constitutively active RhoA in MuSCs revealed a fundamental role of G_12_-G_13_ in integrating signals from different GPCRs to control RhoA activity and MuSC quiescence. Our findings unravel a central role of G_12_-G_13_-RhoA signaling in MuSCs and provide new opportunities for pharmacological manipulation of quiescence.

## Results

### Functional characterization of different GPCRs in quiescent MuSCs

To systematically determine changes in the expression pattern of GPCRs during the transition of MuSC from quiescence to activation, we analyzed five published bulk RNA sequencing (RNA-seq) datasets^15–19^. We identified 39 GPCRs, which showed higher expression in quiescent compared to activated MuSCs in at least two datasets (Supplementary Fig. S1a). Subsequent RT-qPCR analysis revealed substantially higher expression of 19 out of these 39 GPCRs in quiescent compared to activated MuSCs (foldchange > 2) (Supplementary Fig. S1b). Immunofluorescence staining of MuSCs localized on freshly isolated single myofibers confirmed the presence of seven GPCRs in the plasma membrane of quiescent MuSC (Supplementary Fig. S1c, d). We corroborated the enrichment of CalcR and S1P_3_ in quiescent MuSCs^13,14,20^, but also detected GPCRs that have not been described in quiescent MuSC before, such as ET_B_ and NTS_2_ (Supplementary Fig. S1c, d). Localization of the remaining GPCRs could not be validated in the plasma membrane, since suitable antibodies were not available.

**Fig. 1:**
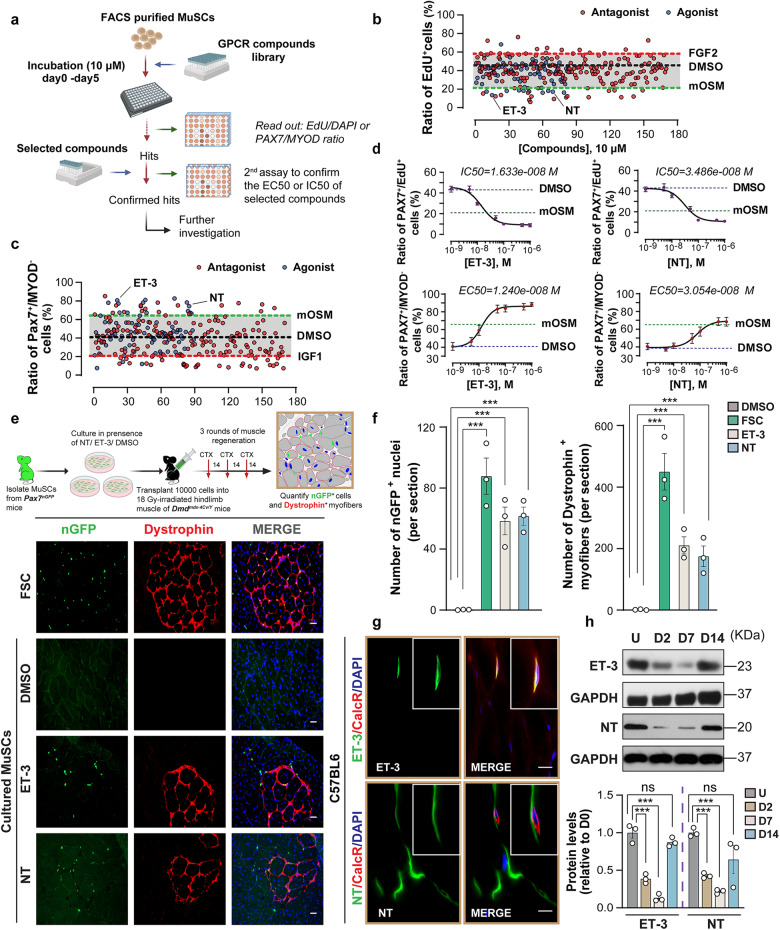
GPCR compound screening identifies ET-3 and NT as regulators of MuSC quiescence. **a** Schematic representation of the GPCR compound screening strategy. **b**, **c** Scatter plots of EdU^+^ (**b**) and PAX7^+^MYOD^‒^ (**c**) MuSC ratios following 5-day treatment with 259 synthetic and natural GPCR compounds (10 μM, GPCR antagonists (red) and agonists (blue)). Green dashed lines: threshold for positive selection (mouse OSM treatment); red dashed lines: negative selection (FGF2 or IGF1 treatment); black lines: DMSO-treated MuSCs. **d** Inhibition of proliferation (upper panel) and myogenic activation (lower panel) of MuSCs by ET-3 and NT (1 nM–1 µM). **e**, **f** Immunofluorescence (**e**) and quantification of GFP^+^ nuclei and Dystrophin^+^ myofibers (**f**) in transverse sections of *Dmd*^*mdx-4Cv/Y*^ TA muscles, engrafted with freshly isolated MuSC (FSC) or cultured in the presence of DMSO, ET-3, and NT for 5 days, after 3 consecutive CTX-induced injuries. The cartoon in **e** represents the schematic outline of MuSC engraftment (*n* = 3). **g** Immunofluorescence for CalcR (red), ET-3/NT (green), and DAPI (blue) on transverse sections of TA muscles. **h** Western blot analysis for ET-3/NT in uninjured (U) TA muscles and injured TA muscles at D2, D7, and D14 after CTX injury. Quantification is shown in the lower panel (*n* = 3). The data represent means ± SEM, analyzed by one-way ANOVA with Bonferroni’s multiple comparisons test (**f**, **h**). Scale bars: 20 µm in **e**, 5 µm in **g**.

Expression of a GPCR does not necessarily prove a physiologically relevant function. Thus, we combined the expression analysis with an unbiased pharmacological screen^21^, utilizing a customized GPCR compound library consisting of 259 compounds, targeting 24 subfamilies of GPCRs, including both natural and chemical agonists and antagonists (Supplementary Fig. S2a). We reasoned that manipulation of GPCRs or ligand‒GPCR pairs, functionally important for inducing or maintaining quiescence, should alter activation of MuSC in vitro. To assess MuSC activation, we performed EdU-incorporation assays and quantified the ratio of quiescent PAX7^+^MYOD^‒^ to all PAX7^+^ cells. (Fig. 1a). Cut-offs for potential hits were defined by activities of Oncostatin M (OSM) (Fig. 1b, c), which induces quiescence of MuSCs^18^, and FGF2 and IGF1 (Fig. 1b, c), which induce proliferation and myogenic differentiation of MuSCs, respectively^22,23^. We identified 15 GPCR subfamilies, whose manipulation changed quiescence or activation of MuSCs, including adrenergic receptors (ARs), Cannabinoid receptors (CB1 and CB2), metabotropic glutamate receptors (mGluRs), and Thyroid-stimulating hormone receptor (TSHR) (Supplementary Fig. S2b). Furthermore, we found that ET-3 and NT prevented expansion and myogenic commitment of MuSC at low dosages (Fig. 1d). Similar results were obtained when MuSCs attached to isolated single myofibers from mouse flexor digitorum brevis (FDB) muscles were treated with ET-3 or NT (Supplementary Fig. S2c, d). The strong activity of ET-3 and NT is consistent with the presence of the corresponding receptors ET_B_ and NTS_2_ in the plasma membrane of quiescent MuSCs (Supplementary Fig. S1c).

We next tested whether ET-3 or NT is able to retain stem cell properties of MuSCs, which are normally lost during expansion in vitro, reducing the ability of expanded MuSC to contribute to skeletal muscle regeneration in a mouse model of Duchenne muscular dystrophy (*Dmd*^*mdx-4Cv/Y*^ mice)^24,25^. MuSCs were isolated from *Pax7nGFP*^*+/+*^ mice, specifically labeling nuclei of PAX7^+^ cells with GFP, and cultured for 5 days with and without the presence of ET-3 or NT. Afterward, cultured MuSCs were grafted into the tibialis anterior (TA) muscle of 18 Gy-irradiated hindlimbs of *Dmd*^*mdx-4Cv/Y*^ mice, shortly after the first three consecutive cardiotoxin (CTX) injections (Fig. 1e). We also used freshly isolated MuSCs, which typically show a higher regenerative capacity than cultured cells^7,26^. Self-renewal and regenerative capacity of transplanted MuSCs were examined by counting the number of GFP-positive nuclei and dystrophin-positive myofibers after completion of regeneration. Treatment with ET-3 or NT enabled transplanted MuSCs to massively increase the numbers of dystrophin-positive myofibers and GFP-positive nuclei within the host tissue compared to untreated controls, although the efficiency of freshly isolated MuSCs was not fully reached (Fig. 1e, f). The results suggested that NT or ET-3 treatment promotes engraftment of cultured MuSC as indicated by higher numbers of Pax7-GFP^+^ cells and also improves the contribution of transplanted MuSC to regenerating muscle fibers in *mdx* muscles.

### ET-3 and NT are niche-derived factors, preventing premature activation of MuSCs in vivo

To investigate the origin of ET-3 and NT from cells in the MuSC niche, we first consulted the “*scmuscle*” database (scRNAseq.org), a recently constructed omics database that provides information about transcript levels in single cells of skeletal muscles under various conditions^27^. According to the *scmuscle* database, expression of *Edn3* (the gene encoding for ET-3) is highest in quiescent MuSCs compared to other cells in the skeletal muscle, implying an autoregulatory loop to keep MuSCs in quiescence (Supplementary Fig. S2e). In contrast, *Nts* (the gene encoding for NT) is mainly found in endothelial cells (Supplementary Fig. S2e), consistent with prior findings^28^. RT-qPCR and immunofluorescence analyses confirmed that *Edn3* is expressed in quiescent but not in activated MuSCs, whereas *Nts* is expressed in lymphatic and blood endothelial cells, directly adjacent to quiescent MuSCs (Fig. 1g; Supplementary Fig. S2f‒i). Specificity of antibodies against NT was verified by western blot analysis of lymph endothelial cells after siRNA-mediated knockdown of NTS (Supplementary Fig. S2j, k). Both ET-3 and NT are strongly downregulated but gradually restored towards the final stages of muscle regeneration (Fig. 1h). Interestingly, we also observed a substantial decline of *Edn3*, *Ednrb*, and *Ntsr2* expression in aged (24-month-old) MuSCs (Supplementary Fig. S2l, n). The expression of ET-3 and NT in the MuSC niche as well as their decline following muscle injury, suggest a role of ET-3 and NT in regulating stem cell quiescence. Similarly, the reduced expression of *Edn3*, *Ednrb*, and *Ntsr2* during aging may contribute to the reduced quiescence of aged MuSCs.

**Fig. 2:**
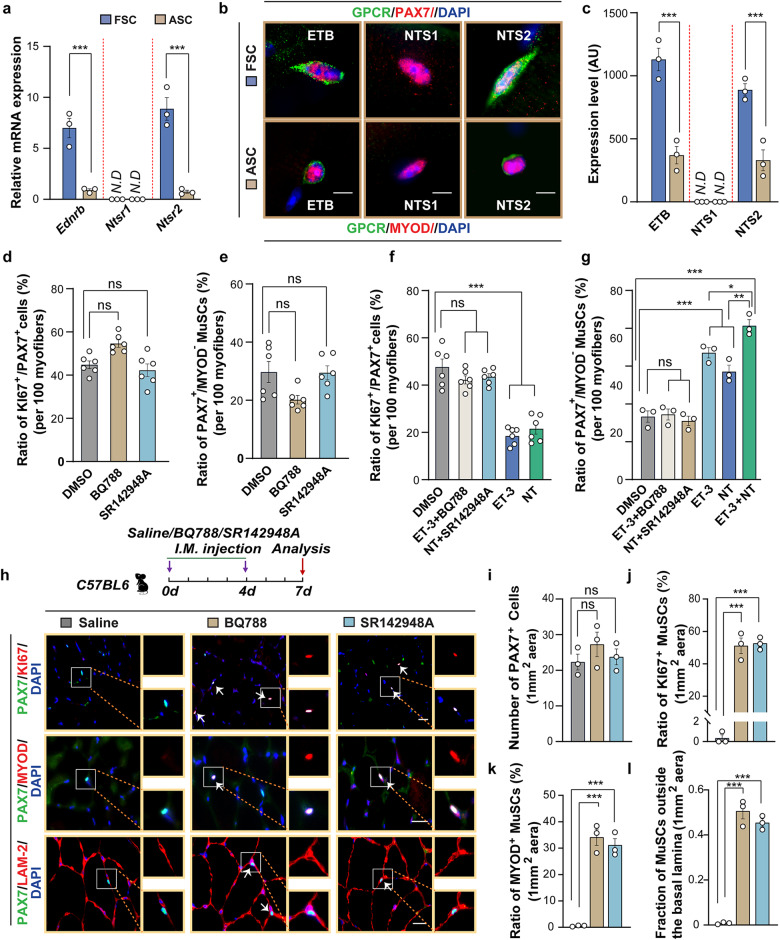
Pro-quiescence effects of ET-3 and NT on MuSCs are mediated by ET_B_ and NTS_2_. **a** RT-qPCR analysis of *Ednrb*, *Ntsr1*, and *Ntsr2* expression in freshly isolated MuSCs (FSC) and activated stem cells (ASC) (*n* = 3). **b** Representative images of immunostaining for ET_B_/NTS_1_/NTS_2_ (red), PAX7 (green) and DAPI (blue) on FDB myofibers of WT mice at 0 h (upper panel) and 24 h (lower panel) of culturing. **c** The quantification of GPCR staining intensity (*n* = 3). **d**, **e** Quantification of the ratios of PAX7^+^KI67^+^ (**d**) and PAX7^+^MYOD^‒^ (**e**) MuSCs on FDB myofibers after 24 h culturing in the presence of DMSO, BQ788 or SR142948A (*n* = 6). **f**, **g** Quantification of the ratio of PAX7^+^KI67^+^ (**f**) and PAX7^+^MYOD^−^ (**g**) MuSCs on FDB myofibers after 24 h culturing in the presence of DMSO, ET-3, NT, ET-3 + BQ788, NT + SR142948A, or ET-3 + NT (*n* = 6). **h**‒**l** Representative images (**h**) and quantification of PAX7^+^ (**i**), the ratio of PAX7^+^KI67^+^ (**j**), and PAX7^+^MYOD^+^ (**k**) cells, and quantification of MuSCs outside the basal limina (**l**) on transverse sections of WT mice TA muscle after intramuscular injection of solvent, BQ788 (1 mg/kg bodyweight), or SR142948A (0.5 mg/kg bodyweight), respectively (*n* = 3). The cartoon in **h** depicts the outline of the experimental design. The data represent means ± SEM, analyzed by unpaired *t*-test (**a**, **c**) and one-way ANOVA with Bonferroni’s multiple comparisons test (**d**‒**g** and **i**‒**l**). ND not detected. Scale bars: 5 µm in **b**, 10 µm in **h**.

Consistent with the results of our initial GPCR expression profiling, subsequent RT-qPCR and immunofluorescence analyses revealed high expression of the receptors for ET-3 and NT, ET_B_ and NTS_2_ in quiescent but not in activated MuSCs (Fig. 2a‒c). We also found that treatment of cultured MuSC with ET-3 and NT increases expression of *Ednrb*, and *Ntsr2*, respectively, indicating the existence of a positive feedback loop, which secures enhanced *Ednrb* and *Ntsr2* expression in MuSCs when regeneration is completed (Supplementary Fig. S2o, p). The second receptor for NT, NTS_1_, is neither expressed in quiescent nor in activated MuSCs (Fig. 2a‒c). To study the roles of ET_B_ and NTS_2_ in the regulation of MuSC quiescence, we utilized selective antagonists for ET_B_ (BQ788) and NTS_2_ (SR142948A). Treatment of MuSCs attached to isolated single FDB myofibers with BQ788 or SR142948A did not change proliferation of MuSCs or the ratio of PAX7^+^MYOD^+^ to all PAX7^+^ MuSCs, probably due to low concentration of ET-3 and NT in the experimental set-up (Fig. 2d, e). However, treatment with BQ788 after prior administration of ET-3, abrogated inhibitory effects of ET-3 on MuSC proliferation and MYOD expression (Fig. 2f, g). Similar consequences were observed when SR142948A was added to the growth medium of single FDB myofibers treated with NT (Fig. 2f, g). Combined treatment with ET-3 and NT further increased the ratio of PAX7^+^MYOD^‒^ relative to all PAX7^+^ MuSCs compared to single treatments, although no synergistic effects were observed. This might be due to the rather artificial experimental set-up, providing higher concentrations of ET-3 and NT than normally available to MuSCs in vivo (Fig. 2g). To validate the roles of ET_B_ and NTS_2_ for maintaining MuSC quiescence in vivo, we injected BQ788 or SR142948A into TA muscles of WT mice. After 7 days of exposure to either BQ788 or SR142948A, 33% of MuSCs in BQ788-treated mice and 65% in NT-treated mice were located outside the basal lamina in the interstitial space, indicating MuSC activation. Likewise, the ratios of KI67^+^PAX7^+^ and MYOD^+^PAX^+^ to all PAX7^+^ cells increased dramatically, whereas the overall numbers of PAX7^+^ cells did not change after treatment with BQ788 or SR142948A (Fig. 2h‒l). We concluded that activation of ET_B_ and NTS_2_ is essential to keep MuSC arrested in quiescence within the niche.

### G_12_-G_13_ integrate signals from different GPCRs to maintain quiescence of MuSCs

**Fig. 3:**
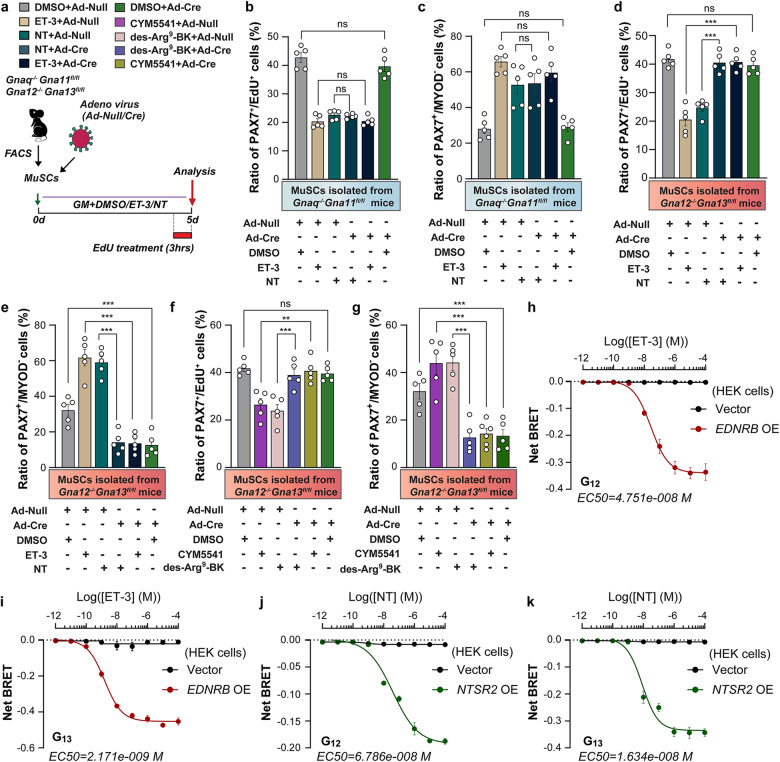
ET-3 and NT prevent MuSC activation through G_12_-G_13_ signaling. **a** Schematic experimental outline of **b**‒**g**. **b**, **c** Ratio of PAX7^+^EdU^+^ (**b**) and PAX7^+^MYOD^‒^ (**c**) MuSCs isolated from *Gnaq*^*‒/‒*^*Gna11*^*fl/fl*^ mice infected with Ad-Null or Ad-Cre virus and cultured in the presence of DMSO, ET-3, or NT for 5 days (*n* = 5). **d**, **e** Ratios of PAX7^+^EdU^+^ (**d**) or PAX7^+^MYOD^‒^ (**e**) MuSCs isolated from *Gna12*^*‒/‒*^*Gna13*^*fl/fl*^ mice infected with Ad-Null or Ad-Cre virus and cultured in the presence of DMSO, ET-3, or NT for 5 days in vitro (*n* = 5). **f**, **g** Ratio of PAX7^+^EdU^+^ (**f**) and PAX7^+^MYOD^‒^ (**g**) MuSCs isolated from *Gna12*^*‒/‒*^*Gna13*^*fl/fl*^ mice infected with Ad-Null or Ad-Cre virus and cultured in the presence of DMSO, CYM5541, or des-Arg^9^-Bradykinin (des-Arg9-BK) for 5 days (*n* = 5). **h**, **i** Response curves to ET-3 for activation of G_12_(134)-RLuc8/Gβ3γ9-GFP2 (**h**) and G_13_(126)-RLuc8/Gβ3γ9-GFP2 (**i**) BRET biosensors in control (vector) or *EDNRB*-overexpressing HEK cells (3 independent experiments, 3 replicates per group). **j**, **k** Response curves to NT for activation of G_12_(134)-RLuc8/Gβ3γ9-GFP2 (**j**) and G_13_(126)-RLuc8/Gβ3γ9-GFP2 (**k**) BRET biosensors in control (vector) or *NTRS2*-overexpressing HEK cells (3 independent experiments, 3 replicates per group). The data represent means ± SEM, analyzed by one-way ANOVA with Bonferroni’s multiple comparisons test (**b**‒**g**).

Upon ligand binding, GPCRs couple to different G-proteins, enabling them to activate various intracellular signaling pathways. Signals from distinct GPCRs may also converge on the same G-protein subtype, which can integrate signaling events initiated by multiple ligands. To investigate which G-protein is employed by NTS_2_ and ET_B_ to promote MuSC quiescence, we focused on G_12_-G_13_ and G_q_-G_11_, since either of these G-proteins has been reported to interact with NTS_2_ and ET_B_ in different physiological processes^29,30^. Because the functions of G_12_ and G_13_ as well as of G_q_ and G_11_ strongly overlap and single inactivation of G_12_, G_13_, G_q_, and G_11_ often does not yield clear effects^12^, we focused on G_12_-G_13_ and G_q_-G_11_ compound mutants. Inactivation of *Gna12*-*Gna13* but not of *Gna11*-*Gnaq* by adenoviral transduction of Cre recombinase into isolated MuSCs from *Gna11*^*‒/‒*^*Gnaq*^*fl/fl*^ and *Gna12*^*‒/‒*^*Gna13* ^*fl/fl*^ mice (Fig. 3a), followed by treatment with ET-3 or NT, strongly increased the ratio of EdU^+^PAX7^+^ to all PAX7^+^ MuSCs (Fig. 3b, d). Accordingly, the number of PAX7^+^MYOD^‒^ to all PAX7^+^ MuSCs declined only in Cre adenovirus-transduced MuSCs from *Gna12*^*‒/‒*^*Gna13*^*fl/fl*^ but not from *Gna11*^*‒/‒*^*Gnaq*^*fl/fl*^ mice (Fig. 3c, e). We also used the same experimental model to investigate potential quiescence-promoting effects of S1P_3_, a GPCR that mediates sphingosine-1-phosphate signaling, and BK_1_, a GPCR that mediates bradykinin signaling. Both GPCRs are enriched in quiescent compared to activated MuSCs. Treatment of MuSCs on isolated myofibers with CYM5541 and des-Arg^9^-Bradykinin (Des-Arg^9^-BK), agonists for S1P_3_ and BK_1_, respectively, reduced the ratios of KI67^+^PAX7^+^ and increased the ratios of PAX7^+^MYOD^‒^ to all PAX7^+^ cells to a similar extent as ET-3 and NT (Supplementary Fig. S3a, b). Likewise, inactivation of G_12_-G_13_ abrogated effects of CYM5541 and Des-Arg^9^-BK on MuSCs (Fig. 3f, g). Since only inactivation of G_12_-G_13_ but not of G_q_-G_11_ abrogated the responsiveness of MuSCs to ET-3 and NT (Fig. 3b‒e), we concluded that G_12_-G_13_ is mandatory for enabling signaling by ET_B_ and NTS_2_, although it is possible that inactivation of G_12_-G_13_ prevents acquisition of quiescence irrespective of specific ligands.

To confirm that activation of ET_B_ or NTS_2_ enhances coupling to G_12_ or G_13_, we employed TRUPATH biosensors, which are optimized bioluminescence resonance energy transfer (BRET2) Gαβγ biosensors^31^. Dissociation of the G_12_ or G_13_ subunits and the Gβγ heterodimer is monitored by measuring the BRET2 signal upon agonist-induced activation of GPCRs. Reduction of the BRET2 signal indicates receptor-mediated G-protein dissociation. Co-expression of full-length ET_B_ or NTS_2_ together with the BRET2 reporters Gβ3, Gγ9-GFP2, and G_12_(134)-RLuc8 or G_13_(126)-RLuc8 in HEK293 cells generated stable BRET2 luminescence signals, which declined in a dose-dependent manner after addition of increasing concentrations of either ET-3 or NT (Fig. 3h‒k). The decline of G_13_-dependent signals was more pronounced and occurred at lower concentrations of ligands compared to G_12_-dependent signals but coupling of ET_B_ and NTS_2_ was evident for both G_12_ and G_13_. Taken together, the results indicate that binding of either ET-3 to ET_B_ or NT to NTS_2_ directly activates G_12_-G_13_ signaling.

### G_12_-G_13_ signaling is indispensable for maintaining quiescence of MuSCs and preventing depletion of the MuSC pool during aging

**Fig. 4:**
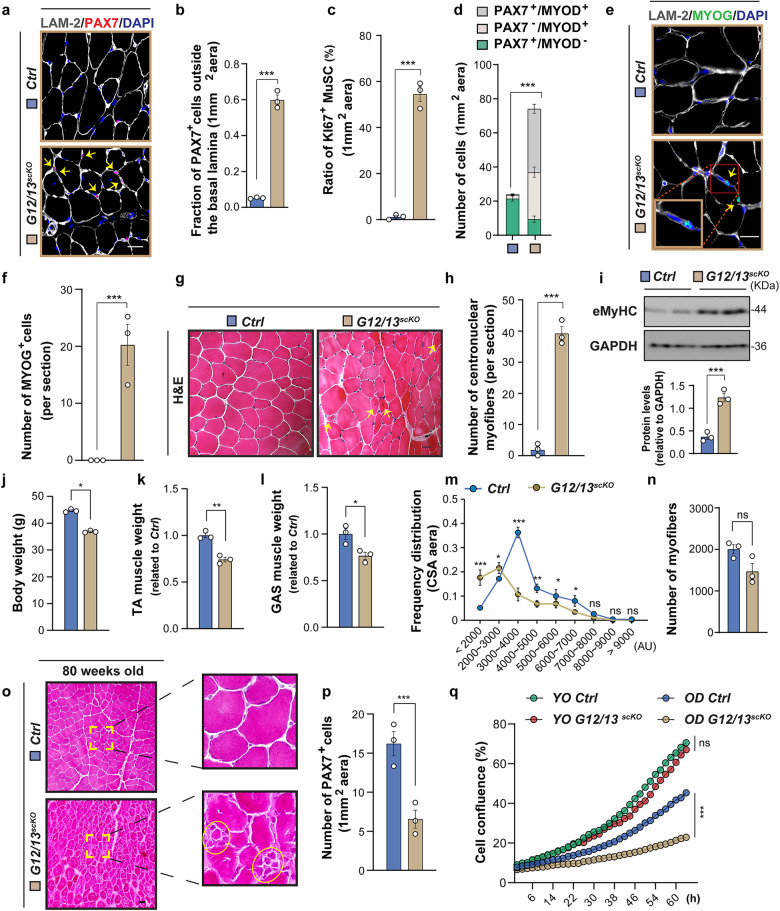
Inactivation of G_12_-G_13_ abrogates MuSC quiescence, depletes the MuSC pool, and enhances sarcopenia during aging. **a**, **b** Immunofluorescence (**a**) and quantification of MuSCs outside the basal lamina (**b**) (*n* = 3). **c** Ratios of KI67^+^ MuSCs in TA muscles of control and *G12/13*^*scKO*^ mice (*n* = 3). **d** Quantification of PAX7^+^MYOD^‒^ (green bars), PAX7^+^MYOD^+^ (gray bars), PAX7^−^MYOD^+^ (ivory bars) cells in TA muscles of control and *G12/13*^*scKO*^ mice (*n* = 3). **e**, **f** Immunofluorescence (**e**) and quantification of MYOG^+^ cells (**f**) in TA muscles of control and *G12/13*^*scKO*^ mice (*n* = 3). **g** H&E staining of TA muscle sections from control and *G12/13*^*scKO*^ mice. **h** Quantification of centronuclear myofibers (*n* = 3). **i** Western blot analysis of eMyHC and GAPDH in TA muscles of control and *G12/13*^*scKO*^ mice (*n* = 3). **j**–**l** Body weight (**j**), TA (**k**) and GAS muscle (**l**) weights of control and *G12/13*^*scKO*^ mice (*n* = 3). **m** Distribution of cross-sectional areas (CSA) of myofibers in TA muscles of control (blue) and *G12/13*^*scKO*^ (yellow) mice (*n* = 3). **n** Quantification of myofibers on transverse sections of aged control and *G12/13*^*scKO*^ TA muscles (*n* = 3). **o** H&E staining of TA muscle sections from control and *G12/13*^*scKO*^ mice. **p** Quantification of PAX7^+^ cells in TA muscles of aged control and *G12/13*^*scKO*^ mice (*n* = 3). **q** Proliferation curve of young (YO, 2-month-old male mice) and old (OD, 80-week-old male mice) control and *G12/13*^*scKO*^ MuSCs (statistical significance pertains to the last time point, *n* = 3). The data represent means ± SEM, analyzed by unpaired *t*-test (**b**‒**d**, **f**, **h**‒**l**, **n** and **p**) and one-way ANOVA with Bonferroni’s multiple comparisons test (**m**, **q**). Male 2-month-old mice were used in **a**‒**i** and 80-week-old male mice in **j**‒**q**. Scale bars: 10 µm in **a**, **e**, 20 µm in **g**, and 50 µm in **o**.

To further examine the role of G_12_-G_13_ as a signaling hub and integrator of GPCR-dependent signaling for inducing quiescence of MuSC, we generated MuSC-specific conditional compound knock-out mice for *Gna12*-*Gna13* (*G12/13*^*scKO*^) (Supplementary Fig. S3c, d). Ten days after initiation of *Gna12*-*Gna13* inactivation, we observed a massive increase of MuSCs, of which 80% were localized outside the basal laminar (Fig. 4a, b). In addition, we detected a major increase of PAX7^+^KI67^+^ double-positive cells in the TA muscles of *G12/13*^*scKO*^ mice, indicating increased activation and proliferation of MuSCs (Fig. 4c; Supplementary Fig. S3e). The numbers of PAX7^+^MYOD^+^ and PAX7^‒^MYOD^+^ were strongly elevated as well, whereas the number of PAX7^+^MYOD^‒^ MuSC declined (Fig. 4d; Supplementary Fig. S3f), indicating reduced self-renewal and enhanced myogenic differentiation. This conclusion was further supported by the presence of numerous MyoG-positive nuclei in *G12/13*^*scKO*^ TA muscles (Fig. 4e, f). Intriguingly, the number of centrally located nuclei surged in *G12/13*^*scKO*^ TA muscles (Fig. 4g, h), associated with increased levels of eMyHC (Fig. 4i), indicating that activation of MuSCs due to depletion of G_12_-G_13_ results in fusion of MuSC to adjacent myofibers or formation of new fibers. This hypothesis was confirmed by genetic lineage tracing, revealing that all centrally located myonuclei are derived from *Gna12*-*Gna13*-deficient mCherry-labeled MuSCs (Supplementary Fig. S3g, h). Numerous mCherry^+^ nuclei were also detected in the periphery of myofibers, demonstrating continuous addition of *Gna12*-*Gna13*-deficient MuSCs and subsequent maturation (Supplementary Fig. S3i). Altogether, these findings demonstrate a critical role of active G_12_-G_13_ signaling for maintaining quiescence of MuSCs. To analyze whether the aberrant activation of *G12/13*^*scKO*^ MuSCs compromises skeletal muscle regeneration and prevents return of mutant MuSCs to the stem cell niche, we subjected *G12/13*^*scKO*^ mice to one- and three-times CTX-induced muscle injury. As expected, *G12/13*^*scKO*^ mice showed signs of compromised muscle regeneration 20 days after the injuries, reflected by fiber size heterogeneity and increased numbers of mononuclear cells (Supplementary Fig. S3j). The numbers of PAX7^+^ MuSCs were substantially lower in *G12/13*^*scKO*^ muscles after completion of regeneration. Moreover, only very few PAX7^+^ MuSCs were detected under the basal lamina, indicating reduced self-renewal and the inability of *G12/13*^*scKO*^ MuSCs to return to the stem cell niche (Supplementary Fig. S3k, l).

In contrast to *G12/13*^*scKO*^ mice, no activation of MuSC was observed in single *G12*^*KO*^ (*Gna12*^*‒/‒*^) and *G13*^*scKO*^ (*Pax7*^*CreERT2*^ *Gna13*^*fl/fl*^) mice, indicating that G_12_ and G_13_ serve overlapping functions in MuSCs (Supplementary Fig. S4a‒e). Similarly, inactivation of *Gnaq-Gna11* in MuSCs did not increase the numbers of PAX7^+^ or PAX7^+^MyoD^+^ MuSCs, the fraction of PAX7^+^ MuSCs outside the basal lamina, or KI67^+^PAX7^+^ cells (Supplementary Fig. S4a‒e). Apparently, G_q_-G_11_ does not play a major role for initiating or maintaining quiescence of MuSC.

To analyze long-term consequences of G_12_-G_13_ depletion and loss of MuSC quiescence, we inactivated *Gna12*-*Gna13* in MuSCs of 2-month-old mice and then allowed the mice to age. At 20 months of age, *G12/13*^*scKO*^ mice showed decreased body weight and muscle mass (Fig. 4j‒l). Aged, 20-month-old *G12/13*^*scKO*^ mice experienced a 26% reduction of TA and a 23% reduction of gastrocnemius (GAS) muscle mass compared to aged control mice (Fig. 4j‒l). Increased loss of muscle mass was associated by disorganized myofiber structures and variations in size, but the numbers of muscle fibers did not decline in statistically significant manner (Fig. 4m‒o).

We also noted changes in fiber-type composition, characterized by a decrease in oxidative type I fibers (Supplementary Fig. S4f, g), different from the consequences of *Gna13* inactivation in myofibers, which increases type 1 and 2a oxidative fibers^32^. Moreover, the number of MuSCs in TA muscles of aged *G12/13*^*scKO*^ dramatically declined (Fig. 4p; Supplementary Fig. S4h), associated with a much lower proliferation rate of aged MuSCs from *G12/13*^*scKO*^ mice compared to MuSCs from control mice (Fig. 4q). The changes in fiber-type composition of *G12/13*^*scKO*^ mice skeletal muscles did not rely on conversion of myofibers into a *G12/13*^*scKO*^ state, which might have been caused by continuous accretion of a *G12/13*^*scKO*^ MuSCs. We only observed a minor reduction of G13 protein in aged muscle fibers of *G12/13*^*scKO*^ mice, even in 80-weeks-old mice (Supplementary Fig. S4i). We also genotyped individual aged muscle fibers from *G12/13*^*scKO*^ mice, reasoning that replacement of existing nuclei from mutant MuSC should result in accumulation of the mutant allele. We clearly detected the genomic fragment derived from the *Gna13* mutant allele in individual myofibers, but the wild-type band was much stronger, indicating that only a subset of myonuclei was replaced or added over time (Supplementary Fig. S4j). These results are well in line with previous reports, demonstrating that myonuclei are rather stable without prior muscle injury^33^. Even in conditions of severe atrophy, the number of myonuclei remain constant without major replacement^34^. Taken together, our findings demonstrate that G_12_-G_13_ signaling is instrumental for maintaining the MuSC pool and ensuring proper MuSC function during aging. Abrogation of G_12_-G_13_ signaling within MuSCs decreases the number of type I myofibers, which is correlated with enhanced skeletal muscle sarcopenia during aging and disrupts normal muscle morphology without loss of G13 protein in aged muscle fibers.

### G_12_-G_13_ signaling in response to ET-3 and NT requires RhoA for suppressing MuSC activation

**Fig. 5:**
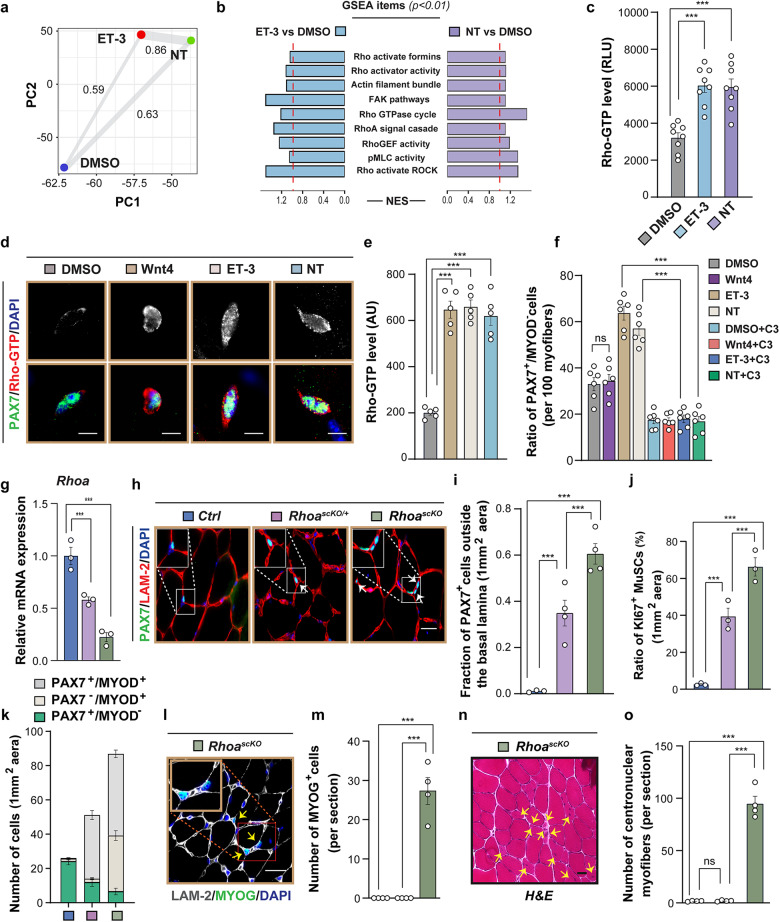
ET-3- and NT-induced G_12_-G_13_ signaling requires RhoA for suppressing MuSC activation. **a** PCA of RNA-seq data from DMSO-, ET-3- and NT-treated MuSCs and Pearson’s values. Each dot represents the mean of three biological samples. **b** GSEA of upregulated and downregulated genes, overlapping between ET-3- or NT-treated MuSCs compared to those treated with DMSO. **c** Rho activity of MuSCs measured by the G-LISA Kit after a 5-day culture with DMSO, ET-3, or NT. Active RhoA-GTP was determined by luminescence at 490 nm (*n* = 8). **d**, **e** Immunofluorescence (**d**) and quantification (**e**) of active Rho in MuSCs on isolated FDB myofibers of WT mice, after 30 min exposure to DMSO, ET-3 or NT (*n* = 5). **f** Ratios of PAX7^+^MYOD^‒^ MuSCs on FDB myofibers from WT mice after 24 h exposure to DMSO, ET-3, or NT, with or without Rho inhibitor (C3) (*n* = 6). **g** RT-qPCR analysis of *Rhoa* expression in fresh isolated MuSCs of control (blue), *Rhoa*^*scKO/+*^ (purple), and *Rhoa*^*scKO*^ (green) mice (*n* = 3). **h**, **i** Immunofluorescence (**h**) and quantification (**i**) of MuSCs outside the basal lamina in TA muscles of control, *Rhoa*^*scKO/+*^, and *Rhoa*^*scKO*^ mice (*n* = 4). **j** Ratios of KI67^+^ MuSCs in TA muscles of control, *Rhoa*^*scKO/+*^ and *Rhoa*^*scKO*^ mice (*n* = 3). **k** Quantification of PAX7^+^ (green bars), PAX7^+^MYOD^+^ (gray bars), and MYOD^+^ (ivory bars) cells in TA muscles of control, *Rhoa*^*scKO/+*^, and *Rhoa*^*scKO*^ mice (*n* = 4). **l**, **m** Immunofluorescence (**l**) and quantification of MYOG^+^ cells (**m**) in TA muscles of *Rhoa*^*scKO*^ mice (*n* = 4). **n**, **o** H&E staining of *Rhoa*^*scKO*^ TA muscles sections (**n**). Quantification of centronuclear myofibers (**o**) in TA muscles of control, *Rhoa*^*scKO/+*^, and *Rhoa*^*scKO*^ mice (*n* = 4). The data represent means ± SEM, analyzed by one-way ANOVA with Bonferroni’s multiple comparisons test (**c**, **e**–**g**, **i**, **j**, **m** and **o**). Scale bars: 5 µm in **d**, 10 µm in **h**, **l** and 20 µm in **n**.

G_12_-G_13_ activate different intracellular signaling pathways, including Jun kinase (JNK) and cyclooxygenase-2 (COX-2), but the main downstream event is direct regulation of RH-RhoGEFs, which activates Rho GTPases^35^. To gain insights into the signaling pathways activated by G_12_-G_13_, we investigated changes in the transcriptional activity of fluorescence-activated cell sorting (FACS)-purified MuSCs treated with ET-3 or NT for 5 days. Principal component analysis (PCA) of RNA-seq data revealed strong differences between treated and non-treated sample but a high correlation between ET-3- or NT-treated MuSCs with an R-value of 0.86 (Fig. 5a). The majorities of upregulated and downregulated genes (69.47% and 79.55%, respectively) were identical between ET-3 and NT treatments, relative to treatment with solvent (Supplementary Fig. S5a). Further Gene Ontology (GO), Kyoto Encyclopedia of Genes and Genomes (KEGG), and Gene Set Enrichment Analysis (GSEA) indicated that upregulated and downregulated genes were primarily associated with Rho signaling pathways, suggesting that members of Rho family are the major effector molecules mediating G_12_-G_13_ signaling after ET-3 or NT stimulation (Fig. 5b; Supplementary Fig. S5b, c). Next, we examined the levels of active-Rho (Rho-GTP) following 5 days of G_12_-G_13_ activation by ET-3 and NT treatment, unraveling a substantial increase of active-Rho levels in MuSCs compared to control (Fig. 5c). Consistent with these findings, we observed a similar upregulation of active-Rho levels in MuSCs on isolated single FDB myofibers after treatment for 30 min with ET-3 or NT. The increase of active-Rho induced by ET-3 or NT was comparable to the effects of WNT4, which has been previously reported to activate RhoA in quiescent MuSCs (Fig. 5d, e). However, unlike ET-3 or NT and despite increased levels of active Rho following WNT4 treatment, WNT4 was unable to limit activation and proliferation of MuSC in vitro (Fig. 5f; Supplementary Fig. S5d). We speculate that effects of WNT4 treatment on RhoA activation is dissenting, less durable, or more indirect compared to ET-3 and NT, although we did not analyze such possibilities in detail. Consistent with these findings, the Rho inactivator C3 transferase (C3) blocked ET-3/NT-induced effects on MuSCs ex vivo (Fig. 5f; Supplementary Fig. S5d), confirming that Rho family members are the pivotal downstream mediators of G_12_-G_13_ signaling induced by ET-3 or NT. Analysis of active-Rho levels in MuSCs from *G12/13*^*scKO*^ mice confirmed the dependency of Rho activation on G_12_-G_13_ signaling. In *Gna12*-*Gna13*-deficient MuSCs on single myofibers isolated of *G12/13*^*scKO*^ mice, we observed significantly lower levels of active Rho and phosphorylated-Myosin Light Chain (pMLC), which is regulated by the Rho/Rho-kinase signaling pathway (Supplementary Fig. S5e‒h).

Twenty mammalian Rho GTPases have been described, of which RhoA is one of the most prominent members, often activated through G_12_-G_13_^36^. To test whether RhoA is indeed the critical mediator of G_12_-G_13_ signaling induced by ET-3 and NT, we conditionally inactivated *Rhoa* in MuSCs using *Rhoa*^*scKO*^ mice, in which gene inactivation is driven by *Pax7-CreERT* after tamoxifen administration (Fig. 5g). Homozygous inactivation of *Rhoa* in MuSCs dramatically increased the number of PAX7^+^ MuSCs outside the stem cell niche (Fig. 5h, i) and the number of proliferating Ki67^+^PAX7^+^ cells 24 days after initiation of gene inactivation (Fig. 5j). Furthermore, Pax7^+^MYOD^‒^ MuSCs declined whereas Pax7^+^MYOD^+^ and Pax7^‒^MYOD^+^ cells increased (Fig. 5k). Heterozygous inactivation of *Rhoa* had similar but far less pronounced effects and showed some notable differences to the homozygous mutant state (Fig. 5h‒j). We did not observe a significant increase of Pax7^‒^MYOD^+^ cells in heterozygous *Rhoa*^*scKO/+*^ muscles (Fig. 5k) and no increase of MYOG-positive muscle cells and centrally located myonuclei, which were abundant in homozygous *Rhoa*^*scKO*^ muscles (Fig. 5i‒o). Apparently, full activation of the myogenic program, indicated by expression of MYOG, resulting in the fusion of MuSCs to adjacent fibers and giving rise to centrally located myonuclei, requires nearly complete repression of RhoA activity. Taken together, inactivation of *Rhoa* in MuSCs fully phenocopies the loss of *Gna12*-*Gna13*, indicating that RhoA is necessary and irreplaceable for relaying G_12_-G_13_-dependent quiescence signals, probably serving as the primary downstream effector of G_12_-G_13_ in MuSCs to secure quiescence.

### Activation of RhoA is sufficient to maintain MuSC quiescence in the absence of G_12_-G_13_ and has no effects on cytoplasmic projections of quiescent MuSC

**Fig. 6:**
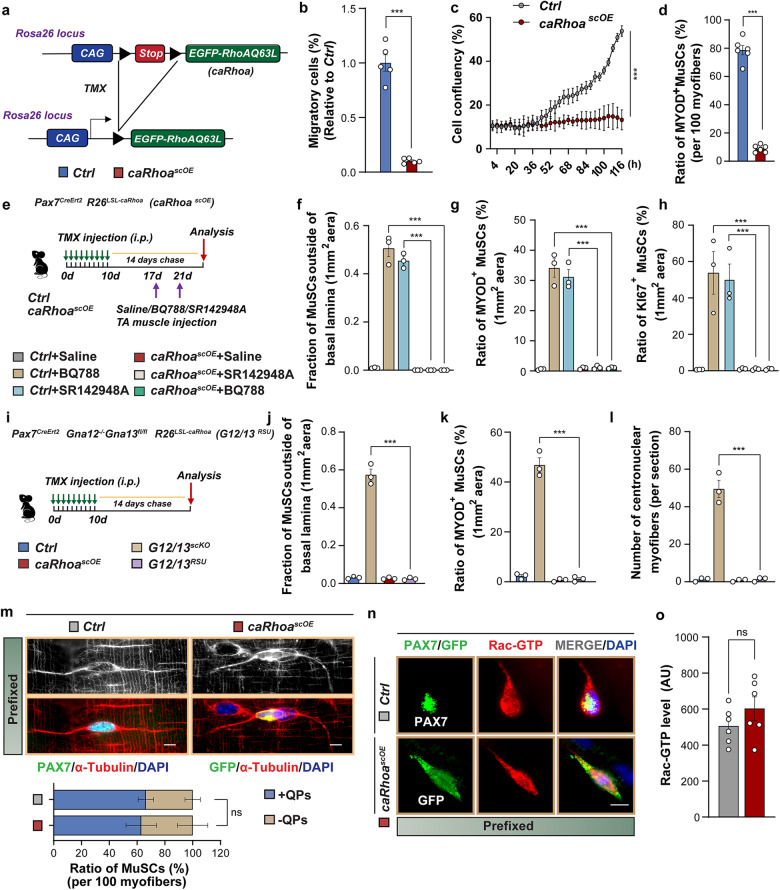
Constitutive activation of RhoA enforces MuSC quiescence and bypasses effects caused by inhibition of G_12_-G_13_ signaling. **a** Design of the *caRhoa* overexpression allele. **b** Motility measurement of control and *caRhoa*^*scOE*^ MuSCs for 5 days in transwell migration assays (*n* = 5). **c** Time-lapse imaging of MuSCs proliferation. Gray dots: control MuSCs; red dots: *caRhoa*^*scOE*^ MuSCs (statistical significance at the last time point, *n* = 3). **d** Ratio of MYOD^+^PAX7^+^ MuSCs on FDB myofibers, 8 h after isolation from control and *caRhoa*^*scOE*^ mice (*n* = 6). **e** Experimental design of **f**‒**h**. **f**‒**h** Quantification of MuSCs outside the basal limina (**f**) and ratios of MYOD^+^ (**g**) and KI67^+^ (**h**) MuSCs in control and *caRhoa*^*scOE*^ mice in TA muscles intramuscularly injected with saline, BQ788 (1 mg/kg bodyweight), or SR142948A (0.5 mg/kg bodyweight) (*n* = 3). **i** Experimental design of **j**‒**l**. **j**‒**l** Quantification of MuSCs outside the basal lamina (**j**), ratio of MYOD^+^ MuSCs (**k**), and number of centronuclear myofibers (**l**) in control, *caRhoa*^*scOE*^, *G12/13*^*scKO*^, and *caRhoa*^scOE^*/G12/13*^*scKO*^ (*G12/13*^*RSU*^) TA muscles (*n* = 3). **m** Immunofluorescence for PAX7 (green), α-TUBULIN (red), DAPI (blue), and GFP (green) for control and *caRhoa*^*scOE*^ EDL myofibers (upper panel). The proportion of MuSCs with or without QPs is shown in the lower panel (*n* = 3). **n**, **o** Immunofluorescence (**n**) and quantification of Rac-GTP (**o**) in MuSCs on EDL myofibers (*n* = 6). The data represent means ± SEM, analyzed by unpaired *t*-test (**b**, **d**, **m**, **o**) and one-way ANOVA with Bonferroni’s multiple comparisons test (**c**, **f**‒**h**, **j**‒**l**, **n**, **o**). Scale bars: 10 µm in **m** and 5 µm in **n**.

To investigate whether RhoA is not only necessary but also sufficient to arrest MuSCs in quiescence, we generated a mouse model enabling conditional, MuSC-specific expression of a constitutively active mutant (Q63L) of human RhoA (*Pax7*^*CreERT2*^; *Tg*: *R26*^*LSL-caRhoa*^). We also fused a GFP-reporter to the N-terminus of caRhoA to facilitate detection (Fig. 6a). The approach was highly effective and specific. Essentially all Pax7^+^ MuSCs showed GFP-fluorescence, without any GFP fluorescence outside Pax7^+^ MuSCs (Supplementary Fig. S6a, b), and active RhoA was strongly increased (Supplementary Fig. S6c). The enhanced RhoA activity strongly suppressed motility of MuSCs, a hallmark of MuSC activation^37^, in a transwell migration assay (Fig. 6b) and essentially annihilated proliferation of freshly isolated MuSCs (Fig. 6c). Furthermore, enhanced RhoA activity prevented activation of MuSC as indicated by the absence of MYOD expression in MuSCs on isolated myofibers from FDB muscles after 8 h of culture (Fig. 6d; Supplementary Fig. S6d).

To analyze whether constitutively active RhoA preserves quiescence even after inhibition of ET-3 and NT signaling, which normally abrogates MuSC quiescence, we administered the ET_B_ and NTS_2_ blockers SR142948A and BQ788, respectively, to TA muscles of *caRhoa*^*scOE*^ mice (Fig. 6e). Expression of caRhoA prevented activation of MuSC induced by SR142948A and BQ788 in control mice, measured by the absence of MuSCs outside the basal lamina, missing increase of MyoD^+^Pax7^+^ MuSCs, and missing increase in proliferating Ki67^+^/Pax7^+^ MuSCs (Fig. 6f‒h; Supplementary Fig. S6g). We also employed the genetic *G12/13*^*scKO*^ mouse model to demonstrate that increased levels of active RhoA are sufficient to arrest MuSC in quiescence, despite the absence of G_12_-G_13_-mediated signals. Analysis of *caRhoa*^*scOE*^/*G12/13*^*scKO*^ triple mutant mice (*G12/13*^*RSU*^ mice) revealed a full rescue of the *G12/13*^*scKO*^ phenotype by expression of caRhoA, completely eliminating premature MuSC activation and normalizing the number of centrally located myonuclei to control levels (Fig. 6i‒l; Supplementary Fig. S6e‒j). We concluded that RhoA is a central signaling hub, in which not only G_12_-G_13_-derived signals but also others are integrated.

A recent report described that a Rac1-to-Rho switch is required for exiting quiescence and for retraction of complex cytoplasmic projections of quiescent MuSC, named quiescent projections (QPs). The authors postulated a cross-regulated equilibrium between Rac1 and Rho, in which increased Rho activity breaks quiescence^38^. The claim that increased Rho/ROCK signaling abrogates quiescence and results in retraction of QPs is clearly in conflict with our findings. We therefore decided to examine the morphology of QPs as a further indicator of MuSC quiescence and analyze RhoA targets including Rac proteins and ROCKs in *caRhoa*^*scOE*^ MuSCs. Immunofluorescence staining of MuSCs for PAX7 and α-Tubulin on isolated single myofibers from control and *caRhoa*^*scOE*^ mice revealed no effects of increased RhoA activity on the formation of QPs, which is consistent with the arrest of *caRhoa*^*scOE*^ MuSCs in quiescence. Neither did the length of QPs change nor the number of MuSCs with visible QPs (Fig. 6m). Moreover, increased expression of active RhoA did not alter the levels of active Rac-GTP, indicating that RhoA does not suppress Rac in MuSCs in vivo (Fig. 6n, o).

**Fig. 7:**
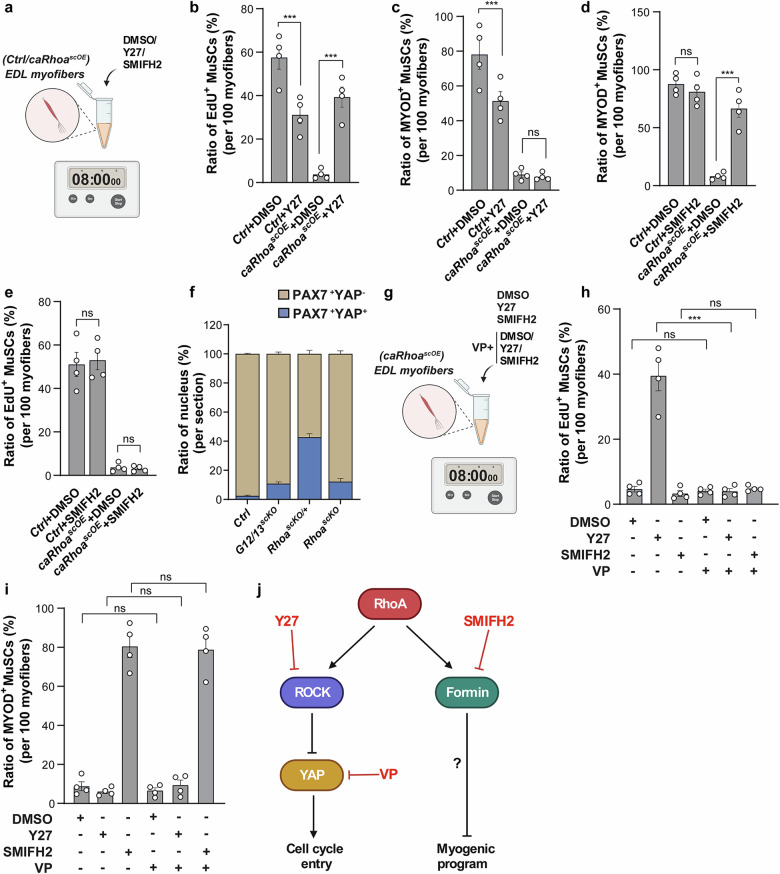
Effects of RhoA to prevent cell-cycle entry and myogenic differentiation are mediated by ROCK1/2-YAP and Formin signaling. **a** Experimental design of **b**‒**e**. **b**, **c** Ratios of EdU^+^ (**b**) and MYOD^+^ (**c**) MuSCs in control and *caRhoa*^*scOE*^ EDL myofibers, following 8-h exposure to DMSO and 10 µM Y27 (*n* = 4). **d**, **e** Ratios of MYOD^+^ (**d**) and EdU^+^ (**e**) MuSCs in control and *caRhoa*^*scOE*^ EDL myofibers following 8-h exposure to DMSO and 20 µM SMIFH2 (*n* = 4). **f** Proportions of YAP^+^ MuSCs on control, *G12/13*^*scKO*^, *Rhoa*^*scKO/+*^ and *Rhoa*^*scKO*^ TA muscle sections (*n* = 3). **g** Schematic representation of the experimental design of **h**, **i**. **h**, **i** Quantifications of the ratio of EdU^+^ (**h**) and MYOD^+^ (**i**) MuSCs in isolated myofibers from FDB muscles of *caRhoa*^*scOE*^ mice after 8 h treatment with DMSO, 10 µM Y27, or 20 µM SMIFH2 alone or in combination with 5 mΜ VP (*n* = 4). **j** Model depicting ROCK-YAP and Formin signaling pathways downstream of RhoA, preventing cell cycle entry and myogenic commitment in quiescent MuSC. The data represent means ± SEM, analyzed by one-way ANOVA with Bonferroni’s multiple comparisons test (**b**‒**e**, **h**, **i**). Drawings displayed in **a**, **g** and **j** were created with BioRender.

After establishing that RhoA does not exert its effects by altering Rac activity, we turned to two well-known targets of RhoA, the Rho-Kinase 1/2 (ROCK1/2) and Diaphanous-related formins (DRFs), which rearrange the cytoskeleton by polymerizing actin^39^. Proliferation of MuSC was measured by EdU incorporation on isolated myofibers and activation of myogenic differentiation by determining the number of MYOD^+^PAX7^+^ cells. Pharmacological inhibition of ROCK1-ROCK2 by the ROCK inhibitor Y-27632 (Y27) released the proliferation block of *caRhoa*^*scOE*^ MuSCs (Fig. 7a, b) but did not release the inhibition of myogenic differentiation, imposed by expression of caRhoA (Fig. 7c). Vice versa, inhibition of DRFs by the general formin inhibitor SMIFH2 induced expression of MYOD in *caRhoa*^*scOE*^ MuSCs on isolated myofibers but had no effects on proliferation (Fig. 7d, e). The differential effects of Y27 and SMIFH2 on MuSCs expressing caRhoA indicate that RhoA controls two different pathways to enforce quiescence, ROCKs for preventing cell cycle entry and formins to suppress myogenic differentiation. Notably, mDia1, a member of the DRF family, has been reported to inhibit expression of MYOD and MYOG, which confirms our findings^40,41^. Since RhoA induces ROCK-mediated phosphorylation of MLC, which suppresses activation and nuclear translocation of YAP in some cell types^8^, we analyzed the presence of YAP in nuclei of control, *G12/13*^*scKO*^, *Rhoa*^*scKO/+*^, and *Rhoa*^*scKO*^ MuSCs. Only very few YAP^+^ nuclei were detected in control MuSCs, whereas deletion of *Gna12-Gna13*, homozygous inactivation of *Rhoa*, and in particular heterozygous inactivation of *Rhoa* raised the number of YAP^+^ nuclei (Fig. 7f). However, we would like to emphasize that a large fraction of MuSCs in *Rhoa*^*scKO*^ and *G12/13*^*scKO*^ mice did not show nuclear translocation of YAP (Fig. 7f), suggesting that RhoA does not solely rely on inhibition of YAP to induce quiescence of MuSCs. To confirm the critical role of YAP downstream of RhoA for stem cell activation, we inhibited ROCK with Y27 and formins with SMIFH2 in *caRhoa*^*scOE*^ MuSCs on isolated myofibers, with or without concomitant treatment with the YAP inhibitor verteporfin (VP) (Fig. 7g). Strikingly, administration of VP abrogated stimulation of EdU incorporation instigated by blockage of ROCK with Y27 but had no effect on MyoD expression, induced by SMIFH2 (Fig. 7h, i). Taken together, these data corroborated the hypothesis that RhoA controls two different pathways to synergistically enforce quiescence, a formin-dependent pathway that inhibits myogenic differentiation and a ROCK-dependent pathway, which inhibits proliferation by suppressing YAP activation (Fig. 7j).

## Discussion

The broad array of signals and mechanisms controlling MuSC quiescence is staggering and hard to understand. Several GPCRs, such as the CalcR and GPR116, are already known to contribute to MuSC quiescence but their individual impact on quiescence appears to be limited, suggesting the existence of combinatorial and partially overlapping signaling pathways^8,9,13,14^. The discovery that G_12_-G_13_ but not G_q_-G_11_ bundle the input of numerous different GPCRs, including ET_B_, NTS_2_, S1P_3_ and BK_1_, to arrest MuSC in quiescence answers the question why different ligand‒receptor combinations have similar effects on MuSCs. However, it still remains a mystery why so many diverse ligands are employed, all resulting in G_12_-G_13_ activation. The answer for this conundrum may reside in the different origins of ligands and the need for graded responses of MuSCs to comply to changes in the microenvironment. Taking ET-3 and NT as an example: NT is derived from endothelial cells whereas ET-3 serves MuSCs in an autoregulatory loop. Altered expression of NT in endothelial cells, which may happen due to changes in the blood flow or during ischemia, provides a direct communication channel between the vasculature and MuSCs. Altered expression of ET-3 in MuSC will modify responsiveness of MuSC to other signals from the niche, including signals from endothelial cells. Eventually, information is computed by G_12_-G_13_ to enhance or lower RhoA activity, allowing proportional responses of MuSCs. Similar principles may apply to other adult stem cells but also during development and in pluripotent stem cells^42^.

We identified RhoA as a critical signaling hub downstream of G_12_-G_13_, which integrates signals from different pathways to control initiation of proliferation via ROCK and YAP, and myogenic differentiation via the DRF formins in quiescent MuSCs. We demonstrated that G_12_-G_13_ are dominant activators of RhoA in quiescent MuSCs, although other pathways have been described to activate RhoA in quiescent MuSCs, such as WNT4, derived from myofibers^8^. However, the effects of WNT4 inactivation in myofibers on MuSC quiescence are relatively weak, which corresponds to our observations that, unlike ET-3 and NT, WNT4 is unable to limit activation and proliferation of MuSC in vitro. WNT4 activates RhoA via VANGL2 in the PCP pathway in some cell types^43^. So far, it has not been investigated whether this mechanism is active in MuSCs and whether heterotrimeric G-proteins contribute to WNT-mediated activation of RhoA. Evidence for an involvement of heterotrimeric G-proteins in WNT-signaling is scarce and an involvement of G_12_-G_13_ has not been described so far, although interactions of Frizzled receptors with Gα subunits have been reported by different groups^44,45^. In our hands, regular levels of WNT4 derived from myofibers were unable to sufficiently activate RhoA in the absence of G_12_-G_13_ for maintaining MuSCs quiescence, which might be seen as a hint for involvement of G_12_-G_13_ in WNT4-mediated activation of RhoA. Yet, the effects of WNT4 on RhoA activation via the PCP pathway might be simply weaker compared to GPCR-G_12_-G_13_-mediated RhoA activation or might require additional, synergistic inputs. It will be possible to distinguish between PCP- and G-protein-dependent signaling downstream of WNT4 in future experiments and analyze whether WNT4 signaling can bypass the block in RhoA activation in *G12/13*^*scKO*^ mice and restore MuSC quiescence.

Our studies uncovered that active RhoA simultaneously employs two different mechanisms to prevent activation of MuSCs: (i) suppression of YAP nuclear translocation via ROCK1/2 and (ii) inhibition of myogenic differentiation via activation of DRF formins, which blocks cell cycle entry and obstructs MyoD expression, respectively. These findings are in full agreement with previous reports, showing that mDia1 (a DRF formin) suppresses expression of MyoD and myogenin^40,41^. Likewise, it has been demonstrated that RhoA inhibits YAP activation via ROCK-mediated MLC phosphorylation in certain cell types^8^. We reason that concomitant activation of both pathways is necessary to arrest MuSC in quiescence, since numerous MuSCs in *Rhoa* ^*scKO*^ and *G12/13*^*scKO*^ mice did not show translocation of YAP into the nucleus, despite widespread activation of MuSCs in these mutants.

Increase of active RhoA had no effects on Rac1 activity and on the number and length of QPs, which are considered to be hallmarks of quiescent MuSCs^38,46^. Our data are in conflict with a recent report, describing a cross-regulated equilibrium between Rac1 and Rho, in which increased Rho activity breaks quiescence and results in retraction of QPs^38^. The conflicting data might be explained by different experimental conditions and/or differential roles of activated RhoA during MuSC activation. RhoA is a principal regulator of the F-actin cytoskeletons, controlling F-actin dynamics when quiescent MuSC extend QPs, but also when they retract extensions^38,47–49^. Quiescent MuSCs are dynamic and exhibit variability in protrusions within their natural environment, probably in response to changes in mechanical force, stiffness, or biochemical cues within the muscle, suggesting that retraction of QPs also happens in quiescent MuSCs when RhoA activity is high^46^. We assume that additional signals are necessary for permanent retraction of QPs, which temporarily take advantage of the role of RhoA as a regulator of F-actin dynamics, independent of its role in regulating MuSC quiescence via ROCK1/2 and DRFs. Additional studies are necessary to unveil details of this fascinating process, focusing on temporal changes of RhoA/Rac1 activities in MuSC in vivo.

We found that loss of MuSC quiescence following inactivation of *Gna12-Gna13* results in depletion of the MuSC pool and is associated with enhanced sarcopenia during aging. The correlation between loss of quiescence enhanced sarcopenia during aging is intriguing, but requires further investigations. Resting MuSCs in geriatric mice lose reversible quiescence by switching to an irreversible pre-senescence state, which is a different condition than mere loss of quiescence^1^. Surprisingly, diphtheria-mediated depletion of MuSC impairs skeletal muscle regeneration but without causing sarcopenia^3^. A similar study demonstrated that despite ablation of MuSCs with > 97% efficiency, the average size of cross-sectional areas of myofibers does not decline in aged mice^4^. Compensatory mechanisms within myofibers seem to allow maintenance of fiber sizes even without the influx of new MuSCs, at least to a certain degree and in the absence of severe injury or other challenges. These studies clearly show that it is not necessarily the reduction or absence of MuSC, which causes sarcopenia during aging. Instead, accretion of constantly activated, defective MuSCs might be responsible. This hypothesis is supported by the necessity for tight quality control of MuSCs by programmed cell death to ensure muscle health^50^, but certainly needs further proof. We are speculating that long-term loss of quiescence may lead to the accumulation of persisting chromatin changes or other defects in MuSCs, which may contribute to fiber atrophy and sarcopenia. Careful analysis of defects in MuSCs of aging *G12/13*^*scKO*^ mice and inhibition of MuSC fusion with myofibers will clarify whether such phenomena occur or not. Alternatively, constantly activated or aberrant MuSCs might exert adverse paracrine effects on myofibers, promoting atrophy^51^.

At present, we do not know whether loss of MuSC quiescence, the observed changes in the fiber type composition of *G12/13*^*scKO*^ mice, or completely different processes are responsible for enhanced sarcopenia during aging. *G12/13*^*scKO*^ show a strong reduction of type I myofibers, which increases the relative abundance of non-oxidative type II fibers. The decline in skeletal muscle mass with aging has been mainly attributed to a reduction in type II muscle fiber size^52^. Thus, it is entirely feasible that enhanced sarcopenia during aging is promoted by fiber type switching in *G12/13*^*scKO*^ muscles. Surprisingly, inactivation of *Gna12-Gna13* in MuSCs reduces the number of type I fibers, whereas inactivation of *Gna13* in myotubes increases type I and IIa oxidative fiber^32^, suggesting involvement of different mechanisms. Accordingly, we did not find a substantial reduction of G13 protein levels in myofibers from *G12/13*^*scKO*^ mice, which was matched by the dominant presence of the intact WT *Gna13* allele in individual myofibers. These results suggest that nuclei from *Gna12-Gna13*-deficient MuSCs in non-regenerating muscles only contribute to a minority of nuclei in myofibers, even in 80-week-old mice, corroborating earlier studies that myonuclei are rather stable under physiological conditions^33^.

Taken together the comprehensive characterization of GPCRs, G-proteins, and downstream effectors regulating MuSC quiescence provides novel means for pharmacological restoration of quiescence. We assume that the continuous decline of *Edn3, Ednrb*, and *Ntsr2* expression in MuSCs during aging, marking an age-associated decline in G_12_-G_13_-transduced GPCR signaling in MuSCs, contributes to the loss of MuSC quiescency and attenuated regeneration in aged individuals^53–55^. Pharmacological enhancement of G_12_-G_13_ and RhoA signaling in MuSC may pave the way to better muscle health during aging.

## Materials and methods

### Animals

The *Gnaq*^*‒/‒*^ (*Gnaq* knockout)*, Gna11*^*fl/fl*^*, Gna12*^*‒/‒*^ (*Gna12* knockout)*, Gna13*^*fl/fl*^, and *Rhoa*^*fl/fl*^ mouse strains have been described previously^56–60^. The *Pax7*^*CreERT2*^ mouse strain was obtained from Kardon lab^61^. For engraftment assays, 2-month-old male *Dmd*^*mdx-4Cv/Y*^ mice and male *Pax7*^*nGFP*^ transgenic mice were used^62,63^. Transgenic *R26*^*LSL-caRhoa*^ mice (*Tg: Rosa26 locus: Loxp-stop-Loxp-caRhoa*), expressing a constitutively activate mutant (Q63L) of human RhoA protein with an N-terminal fusion of enhanced GFP (caRhoA) after Cre recombinase-mediated excision of a *loxP-stop-loxP* sequence, were generated by inserting a cassette derived from a plasmid construct (pcDNA3) containing cytomegalovirus enhancer, cytomegalovirus promoter, promotor for bacteriophage T7 RNA polymerase, and a bovine globin polyadenylation signal (# 12968, Addgene) into the *ROSA26* locus of V6.5 mouse embryonic stem (ES) cells. Chimeric mice were generated by injection of ES cells into mouse blastocytes as described^64^. All mice were backcrossed into the C57BL6 genetic background for at least 3 generations. The Primers used for genotyping are shown in Supplementary Table S2.

### Animal procedures

2-month-old male mice were used in all experiments, unless specified otherwise. Intraperitoneal administration of tamoxifen (Sigma) at a dosage of 0.05 mg/g of body weight was carried out on the mice. TA muscles were injected with CTX (0.06 mg/mL, Sigma) in a volume of 50 μL. Engraftment of MuSC was done according to a published protocol^65^. Prior to engraftment, donor cells were counted using a haemocytometer, excluding non-viable cells stained with 0.4% Trypan Blue (Gibco). Donor MuSCs were then centrifuged at 1200× *g*, 4 °C for 20 min and resuspended in DMEM media (Life Technologies) before transplantation into the TA muscle using a 5-µL microcapillary pipette (Drummond). All animal care and handling procedures were conducted in strict accordance with the “Guide for the Care and Use of Laboratory Animals” published by the US National Institutes of Health. The study protocol was approved by the Animal Rights Protection Committee of the State of Hessen (Darmstadt, Germany, approval number: B2/1224), as well as the Institutional Animal Care and Use Committee of Sichuan Agricultural University (Chengdu, China, approval number: 20190241).

### MuSC isolation & culturing

MuSCs were purified via FACS using a published method^6^. Isolated MuSCs were cultured in growth medium 1 (GM1: DMEM-GlutaMAX containing 20% fetal calf serum (FCS), 1% Penicillin/Streptomycin, and 5 ng/mL basic fibroblast growth factor (bFGF)) or in screening medium (SM: DMEM-GlutaMAX containing 20% knockout serum (KSR, Giboc), 1%, Penicillin/Streptomycin, and 1% chicken embryo extract). MuSCs were cultured ± ET-3 (1 μM), ± NT (1.5 μM), ± BQ788 (10 nM), ± SR142948A (5 nM), ± Cell Permeable Rho Inhibitor (C3 Transferase) (1 μg/mL) and other compounds listed in the Supplementary Table S1. GM or SM was supplemented by addition of EdU to a final concentration of 10 μM, 3 h before fixation. Fixed MuSCs were analyzed using the Click-iT EdU kit (Invitrogen) following the manufacturer’s protocol. Time-lapse imaging and analysis were performed using an Incucyte Live-Cell Imaging System and software (Essen Instruments).

### Adenovirus-mediated Cre deletion of floxed sequences in MuSC

FACS-purified MuSCs from *Gna12*^*‒/‒*^*Gna13*^*fl/fl*^ or *Gna11*^*‒/‒*^*Gnaq*^*fl/fl*^ mice were cultured in SM and exposed to Ad-Cre virus (Vector Biolabs) for at least 2 days to facilitate Cre-mediated deletion of Gna13 or Gnaq in MuSCs in vitro. MuSCs treated with adenoviruses that do not express Cre (Ad-Null) served as controls.

### Single myofiber isolation & culturing

Myofibers were obtained through enzymatic digestion of FBD or extensor digitorum longus (EDL) muscles using collagenase 2 (0.02%, Roche) without or with prefixation (mice were perfused with 0.5% paraformaldehyde (PFA), 3 times within 20 min). Unfixed freshly isolated myofibers were cultured in growth medium 2 (GM2: DMEM-GlutaMAX medium supplemented with 20% FCS and bFGF at a concentration of 5 ng/mL) or screening medium 2 (SM2: DMEM-GlutaMAX containing 20% KSR, 1% Penicillin/Streptomycin). Drugs listed in Supplementary Table S1 were added immediately after muscle excision already in the digestion tubes and in culture plates, ensuring immediate exposure. Myofibers were cultured for 0, 8, or 24 h in the absence or presence of these drugs. Subsequently, myofibers were fixed with 4% PFA for 10 mins in preparation for further analysis.

### GPCR compounds screening

For screening of the customized GPCR compound library (Supplementary Table S3), freshly isolated MuSCs were suspended in the screening medium (SM), detailed in the ‘MuSC Isolation & Culturing’ section. 50 µL of the suspension, containing 400 cells, were added into a single well of a 384-well plate (781097, Greiner), precoated with Matrigel (354234, Corning). After seeding, additional 50 µL of SM, containing either the respective compound, known activators or inhibitors (serving as positive and negative controls, respectively), or DMSO (solvent control; final concentration of 0.5%) were added to the wells. Plates were incubated at 37 °C and 5% CO_2_. The different compounds were kept in 10 mM stock solutions in DMSO, ranging from 1%‒100% in water. Compounds were diluted in SM to ensure the final DMSO concentration remained below 0.5% per well. Based on cytotoxicity measurements in a 1‒100 µM range, an initial concentration of 10 µM was selected for primary screening. EdU was added at the fifth day after compound administration to reach a final concentration of 10 μM, three hours before cell fixation. The ratios of EdU^+^/DAPI^+^ and MYOD^+^/PAX7^+^ nuclei were used as a read-out to determine activation of MuSC. Cut-offs for potential hits were defined by the activities of OSM, which induces MuSC quiescence, and FGF2 and IGF1, which promote proliferation and myogenic differentiation of MuSCs. Compounds that showed promise were used for a secondary assay at reduced concentrations.

### Immunofluorescence assay

Myofibers, cryosections, or cultured MuSCs were fixated using 4% PFA for 10 min. Samples were permeabilized, following fixation, using 0.1% Triton X-100 for 15 min. Subsequently, specimens were blocked in a 3% bovine serum albumin (BSA) solution for 30 min and then exposed to the respective antibodies (Supplementary Table S1) overnight at 4 °C. Visualization of immunofluorescence signals was achieved by secondary antibodies conjugated with Alexa488 or Alexa594. A fluorescence microscope (AXIO observer Z1, Zeiss) equipped with objective lenses of 63×, 40×, and 20× was employed to detect and record the fluorescence signals. To quantify fluorescence intensity, ImageJ software was utilized, enabling subsequent statistical analyses of data. Specificity of the antibody against NT (GTX37368, GeneTex) was verified in in vitro cultured primary murine lymphatic endothelial cells after siRNA-mediated knockdown of *Nts* (Supplementary Fig. S2j, k).

### RhoA G-LISA assay

To determine RhoA activity in cultured MuSCs, the RhoA G-LISA Activation Assay kit (BK121, Cytoskeleton) was employed, which measures Rho-GTP level. Cultured MuSCs were starved in serum-free DMEM-GlutaMAX medium for a duration of 10‒30 min. Subsequently, they were stimulated with either ET-3 (3 μM) or NT (3 μM) for 10 min. Following stimulation, cells were lysed with the lysis buffer included in the kit for 10 min on ice. Total cell extracts were prepared and adjusted to a protein concentration of 1 mg/mL for quantitative detection of active and total RhoA according to the manufacturer’s instructions. Luminescence intensity recorded using the LB940 Mithras plate reader (Berthold Technologies) at 490 nm.

### In situ binding assays for Rho & Rac GTPase activity

Fixed myofibers (see above) were blocked in 3% BSA and incubated for 1 h at room temperature with GST-tagged PAK-P21 binding domain (Cytoskeleton Inc., PAK01) proteins and GST-tagged Rhotekin-Rho binding domain (Cytoskeleton Inc., RT01) which specifically binds to active Rac/Cdc42 and active Rho, respectively. Myofibers were washed 3 times with PBS and then incubated with a fluorophore-conjugated anti-GST antibody (Invitrogen) for an hour at room temperature. DAPI (Life Technologies) was utilized to stain nuclei.

### BRET2 assays

HEK 293 T cells were transfected with a plasmid cocktail (1:1:1:1 plasmid DNA ratio of receptor:Gα12(134)-RLuc8/Gα13(126)-RLuc8:Gβ3:Gγ9-GFP2) after reaching a density of 600,000 ~ 800,000 cells per well in 6-well plates following a previously published protocol^31^. Medium of transfected cells was changed daily for 3 days, using selection medium (DMEM containing 10% FCS, 1% Penicillin/Streptomycin, and 200 μg/mL G418). The whole length cDNA of human *EDNRB* and *NTSR2* genes were cloned from EDNRB-Tango (# 66458, Addgene) and NTSR2-Tango (# 66458, Addgene) plasmids, respectively, and inserted into the pcDNA3.1(+) vector subsequently. Selected cells were seeded into 96-well plates, after which the culture medium was replaced with 60 µL of assay buffer (1× Hank’s balanced salt solution (HBSS) + 20 mM HEPES, pH 7.4), followed by addition of 10 µL freshly prepared 50 µM coelenterazine 400a (Nanolight Technologies). After 5 min equilibration, cells were treated with 30 µL of ET-3 or NT for an additional 5 min. Next, luminescence signals were recorded at 395 nm (RLuc8-coelenterazine 400a) and 510 nm (GFP2) emission filters, at integration times of 1 s per well, using the LB940 Mithras plate reader (Berthold Technologies). Fluorescence signals were serially recorded for six times. Measurements from the sixth read were used in all analyses. BRET2 ratios were computed as the ratio of GFP2 to RLuc8 emission. To confirm the specificity of ligand‒receptor interactions in BRET assays, NTSR2-expressing cells were treated with 30 µL of ET-3, and EDNRB-expressing cells with 30 µL of NT. The absence of significant BRET signals in the control groups confirmed the specificity of interactions.

### RNA-seq

RNA-seq analysis was conducted following established protocols^66^. Total RNA was extracted using the RNAeasy Mini kit (QIAGEN), following the manufacturer’s guidelines. Quality and integrity of RNA and of library preparations were assessed using the LabChip Gx Touch 24 (PerkinElmer). For VAHTS Stranded mRNA-seq, 300 ng of total RNA was used as input. Library preparations were performed according to the manufacturer’s protocol (Vazyme). Sequencing was carried out on a NextSeq500 instrument (Illumina) with v2 chemistry, resulting in an average of 30 M reads per library using 1× 75 bp single-end reads. Raw reads underwent quality assessment, adapter content analysis, and duplication rate estimation using FastQC 0.10.1. Trimming of reads was performed using Reaper version 13-100. Subsequently, reads were aligned to the Ensemble mouse genome version mm10 (GRCm38) using STAR 2.4.0a. The featureCounts 1.4.5-p1 tool from the Subread package was employed to count reads aligning to genes. Only reads mapping within exons were considered, and counts were aggregated per gene. Reads overlapping multiple genes or aligning to multiple regions were excluded. DESeq2 version 1.62 l was utilized to identify differentially expressed genes (DEGs). Genes were considered significantly differentially expressed if they exhibited an absolute fold change of ≥ 2, a Benjamini-Hochberg corrected *P* ≤ 0.05, and a minimum combined mean of 5 reads. The “plotPCA function” of R package “DESeq2” was employed to perform PCA on the rlog-transformed count data. Pearson correlation was calculated between the means of CPM normalized expression for each replicate group. Differences in expression were assessed using the lfcShrink function with the apeglm method (v1.6.0). GSEA was performed to characterize DEGs, utilizing gene sets extracted from MSigDB. GO and KEGG analyses were performed using the “clusterProfiler” package of R.

### Western blot assay

Freshly isolated or cultured MuSCs were rinsed with PBS and lysed in cell lysis buffer for 10 mins. Lysates were sonicated and proteins were separated through SDS-PAGE and transferred onto nitrocellulose membranes (Millipore, Billerica, MA). The membranes were probed with primary antibodies detecting G_12_, G_13_, Histone-3 (H3), eMyHC, ET-3, NT and GAPDH using antibodies listed in the Supplementary Table S1. After overnight incubation at 4 °C, HRP-conjugated secondary antibodies and the ECL detection system (Pierce) were used for signal visualization. Protein quantification was performed using ImageJ software.

### RT-qPCR assays

RT-qPCR analyses were performed using SYBR Green or TaqMan probe-based RT-qPCR assays. Total RNA of samples was isolated using RNAeasy Mini kit (QIAGEN), following the manufacturer’s protocols. cDNA was generated with the PrimeScript™ II 1st strand cDNA Synthesis Kit (Takara). TaqMan probe-based RT-qPCR was performed with the TaqMan Fast Advanced Master Mix (Thermo Fisher). The assay IDs of commercial TaqMan probes for detection of genes of interest are listed in Supplementary Table S2. SYBR Green-based RT-qPCR was performed with the Applied Biosystems™ PowerUp™ SYBR™ Green Master Mix (Thermo Fisher). Sequences of primers are listed in Supplementary Table S2. Relative expression levels of genes of interest were determined using the comparative threshold method, using *Gapdh* as an internal control. Data were analyzed using the ΔΔCt method.

### Histological analysis

Muscles were collected at specified time-points, as indicated in each figure, and rapidly frozen by immersion in isopentane cooled with liquid nitrogen. Subsequently, 8 μm thick sections of muscle samples were generated using a cryomicrotome and subjected to Hematoxylin and Eosin (H&E) staining following established protocols.

### Statistics

Statistical information related to individual experiments is provided in the corresponding figure legends. Data sets with sample sizes (*n*) of three or greater were subjected to the Shapiro–Wilk test to assess normality; all data sets presented herein passed this test. For comparison of multiple groups, we used one-way ANOVA and for comparison of two groups, the unpaired two-tailed Student’s *t*-test was used. A *P* < 0.05 was considered to be significant (ns: no significant, *P < 0.05, **P < 0.01, ***P < 0.001). Results are presented as means ± SEM. Data analysis was performed using GraphPad Prism 9 software.

### Supplementary information

Supplementary figures

Supplementary Material, Supplementary Tables

## Data Availability

This paper does not report original code, but image analysis pipelines will be shared by the lead contact upon request. RNA-seq data were deposited in the NCBI Gene Expression Omnibus (GEO) database with the accession number GSE234590. Any additional information required to reanalyze the data reported in this paper is available from the lead contact upon request.

## References

[1] Sousa-Victor P (2014). Geriatric muscle stem cells switch reversible quiescence into senescence. Nature.

[2] Relaix F (2021). Perspectives on skeletal muscle stem cells. Nat. Commun..

[3] Fry CS (2015). Inducible depletion of satellite cells in adult, sedentary mice impairs muscle regenerative capacity without affecting sarcopenia. Nat. Med.

[4] Keefe AC (2015). Muscle stem cells contribute to myofibres in sedentary adult mice. Nat. Commun..

[5] Crist CG, Montarras D, Buckingham M (2012). Muscle satellite cells are primed for myogenesis but maintain quiescence with sequestration of Myf5 mRNA targeted by microRNA-31 in mRNP granules. Cell Stem Cell.

[6] Gunther S (2013). Myf5-positive satellite cells contribute to Pax7-dependent long-term maintenance of adult muscle stem cells. Cell Stem Cell.

[7] Zismanov V (2016). Phosphorylation of eIF2alpha is a translational control mechanism regulating muscle stem cell quiescence and self-renewal. Cell Stem Cell.

[8] Eliazer S (2019). Wnt4 from the niche controls the mechano-properties and quiescent state of muscle stem cells. Cell Stem Cell.

[9] Baghdadi MB (2018). Reciprocal signalling by Notch-Collagen V-CALCR retains muscle stem cells in their niche. Nature.

[10] Hicks MR, Pyle AD (2022). The emergence of the stem cell niche. Trends Cell Biol.

[11] Hayat R, Manzoor M, Hussain A (2022). Wnt signaling pathway: a comprehensive review. Cell Biol. Int..

[12] Wettschureck N, Offermanns S (2005). Mammalian G proteins and their cell type specific functions. Physiol. Rev..

[13] Yamaguchi M (2015). Calcitonin receptor signaling inhibits muscle stem cells from escaping the quiescent state and the niche. Cell Rep..

[14] Sénéchal C (2022). The adhesion G-protein-coupled receptor Gpr116 is essential to maintain the skeletal muscle stem cell pool. Cell Rep..

[15] Liu L (2013). Chromatin modifications as determinants of muscle stem cell quiescence and chronological aging. Cell Rep..

[16] Fukada S (2007). Molecular signature of quiescent satellite cells in adult skeletal muscle. Stem Cells.

[17] L’Honoré A (2018). The role of Pitx2 and Pitx3 in muscle stem cells gives new insights into P38α MAP kinase and redox regulation of muscle regeneration. Elife.

[18] Sampath SC (2018). Induction of muscle stem cell quiescence by the secreted niche factor Oncostatin M. Nat. Commun..

[19] García-Prat L (2016). Autophagy maintains stemness by preventing senescence. Nature.

[20] Fortier M, Figeac N, White RB, Knopp P, Zammit PS (2013). Sphingosine-1-phosphate receptor 3 influences cell cycle progression in muscle satellite cells. Dev. Biol..

[21] Zhang R, Xie X (2012). Tools for GPCR drug discovery. Acta Pharm. Sin..

[22] Engert JC, Berglund EB, Rosenthal N (1996). Proliferation precedes differentiation in IGF-I-stimulated myogenesis. J. Cell Biol..

[23] Yablonka-Reuveni Z, Rivera AJ (1997). Proliferative dynamics and the role of FGF2 during myogenesis of rat satellite cells on isolated fibers. Basic Appl. Myol..

[24] Gilbert PM (2010). Substrate elasticity regulates skeletal muscle stem cell self-renewal in culture. Science.

[25] Montarras D (2005). Direct isolation of satellite cells for skeletal muscle regeneration. Science.

[26] Sacco A, Doyonnas R, Kraft P, Vitorovic S, Blau HM (2008). Self-renewal and expansion of single transplanted muscle stem cells. Nature.

[27] McKellar DW (2021). Large-scale integration of single-cell transcriptomic data captures transitional progenitor states in mouse skeletal muscle regeneration. Commun. Biol..

[28] Li J (2021). Neurotensin is an anti-thermogenic peptide produced by lymphatic endothelial cells. Cell Metab..

[29] Liu B, Wu D (2003). The first inner loop of endothelin receptor type B is necessary for specific coupling to Galpha 13. J. Biol. Chem..

[30] Pelaprat D (2006). Interactions between neurotensin receptors and G proteins. Peptides.

[31] Olsen RHJ (2020). TRUPATH, an open-source biosensor platform for interrogating the GPCR transducerome. Nat. Chem. Biol..

[32] Koo JH (2017). Galpha13 ablation reprograms myofibers to oxidative phenotype and enhances whole-body metabolism. J. Clin. Invest..

[33] McLoon LK, Rowe J, Wirtschafter J, McCormick KM (2004). Continuous myofiber remodeling in uninjured extraocular myofibers: myonuclear turnover and evidence for apoptosis. Muscle Nerve.

[34] Bruusgaard JC, Gundersen K (2008). In vivo time-lapse microscopy reveals no loss of murine myonuclei during weeks of muscle atrophy. J. Clin. Invest..

[35] Yang YM, Kuen DS, Chung Y, Kurose H, Kim SG (2020). Galpha(12/13) signaling in metabolic diseases. Exp. Mol. Med..

[36] Etienne-Manneville S, Hall A (2002). Rho GTPases in cell biology. Nature.

[37] Baghdadi MB (2018). Notch-induced miR-708 antagonizes satellite cell migration and maintains quiescence. Cell Stem Cell.

[38] Kann AP (2022). An injury-responsive Rac-to-Rho GTPase switch drives activation of muscle stem cells through rapid cytoskeletal remodeling. Cell Stem Cell.

[39] Kühn S, Geyer M (2014). Formins as effector proteins of Rho GTPases. Small GTPases.

[40] Saleh A, Subramaniam G, Raychaudhuri S, Dhawan J (2019). Cytoplasmic sequestration of the RhoA effector mDiaphanous1 by Prohibitin2 promotes muscle differentiation. Sci. Rep..

[41] Gopinath SD, Narumiya S, Dhawan J (2007). The RhoA effector mDiaphanous regulates MyoD expression and cell cycle progression via SRF-dependent and SRF-independent pathways. J. Cell Sci..

[42] Doze VA, Perez DM (2013). GPCRs in stem cell function. Prog. Mol. Biol. Transl. Sci..

[43] Dreyer, C. A., VanderVorst, K., Carraway, K. L. Vangl as a master scaffold for Wnt/Planar cell polarity signaling in development and disease. *Front. Cell Dev. Biol.* **10**, (2022). 10.3389/fcell.2022.887100 PMC9130715 35646914

[44] Nichols AS, Floyd DH, Bruinsma SP, Narzinski K, Baranski TJ (2013). Frizzled receptors signal through G proteins. Cell Signal..

[45] Wright SC (2019). A conserved molecular switch in Class F receptors regulates receptor activation and pathway selection. Nat. Commun..

[46] Ma N (2022). Piezo1 regulates the regenerative capacity of skeletal muscles via orchestration of stem cell morphological states. Sci. Adv..

[47] Schaks M, Giannone G, Rottner K (2019). Actin dynamics in cell migration. Essays Biochem..

[48] Ridley AJ (2015). Rho GTPase signalling in cell migration. Curr. Opin. Cell Biol..

[49] Sit S-T, Manser E (2011). Rho GTPases and their role in organizing the actin cytoskeleton. J. Cell Sci..

[50] Sreenivasan K (2020). Attenuated epigenetic suppression of muscle stem cell necroptosis is required for efficient regeneration of dystrophic muscles. Cell Rep..

[51] Li L, Xiong WC, Mei L (2018). Neuromuscular junction formation, aging, and disorders. Annu. Rev. Physiol..

[52] Nilwik R (2013). The decline in skeletal muscle mass with aging is mainly attributed to a reduction in type II muscle fiber size. Exp. Gerontol..

[53] Arai, F., Suda, T. Quiescent stem cells in the niche. In *StemBook.* Cambridge: Harvard Stem Cell Institute (2008). 20614597

[54] Chakkalakal JV, Jones KM, Basson MA, Brack AS (2012). The aged niche disrupts muscle stem cell quiescence. Nature.

[55] Blau HM, Cosgrove BD, Ho AT (2015). The central role of muscle stem cells in regenerative failure with aging. Nat. Med..

[56] Wettschureck N (2001). Absence of pressure overload induced myocardial hypertrophy after conditional inactivation of Galphaq/Galpha11 in cardiomyocytes. Nat. Med..

[57] Offermanns S (1998). Embryonic cardiomyocyte hypoplasia and craniofacial defects in G alpha(q)/G alpha(11)-mutant mice. EMBO J..

[58] Gu JL, Müller S, Mancino V, Offermanns S, Simon MI (2002). Interaction of Gα12 with Gα13 and Gαq signaling pathways. Proc. Natl. Acad. Sci..

[59] Moers A (2003). G13 is an essential mediator of platelet activation in hemostasis and thrombosis. Nat. Med..

[60] Melendez J (2011). RhoA GTPase is dispensable for actomyosin regulation but is essential for mitosis in primary mouse embryonic fibroblasts*. J. Biol. Chem..

[61] Murphy MM, Lawson JA, Mathew SJ, Hutcheson DA, Kardon G (2011). Satellite cells, connective tissue fibroblasts and their interactions are crucial for muscle regeneration. Development.

[62] Sambasivan R (2009). Distinct regulatory cascades govern extraocular and pharyngeal arch muscle progenitor cell fates. Dev. Cell.

[63] Sicinski P (1989). The molecular-basis of muscular-dystrophy in the mdx mouse—a point mutation. Science.

[64] Du YB, Xie W, Zhang F, Liu CY (2019). Chimeric mouse generation by ES cell blastocyst microinjection and uterine transfer. Methods Mol. Biol..

[65] Angelino E (2018). Mouse satellite cell isolation and transplantation. Bio-Protoc..

[66] Zhang T (2015). Prmt5 is a regulator of muscle stem cell expansion in adult mice. Nat. Commun..

