# Supplementary material for: RhoA-mediated G12-G13 signaling maintains muscle stem cell quiescence and prevents stem cell loss
Source: Cell Discov. 2024 Jul 16;10:76. doi: 10.1038/s41421-024-00696-7 (PMC11251043; doi:10.1038/s41421-024-00696-7)
Supplement: Supplementary file 1 — Supplementary figures [file 41421_2024_696_MOESM1_ESM.pdf]

Supplementary information

RhoA-mediated G_{12}-G_{13} signaling maintains muscle stem cell quiescence and prevents stem cell loss

Yundong Peng[1#*], Jingjing Du[#1], Rui Li[2], Stefan Günther[1], Nina Wettschureck[2, 3], Stefan Offermanns[2, 3], Yan Wang[4], Andre Schneider[1] and Thomas Braun[1,3*]

*Corresponding authors:

Yundong Peng (Yundong.Peng@mpi-bn.mpg.de)

Thomas Braun (thomas.braun@mpi-bn.mpg.de); (Lead Contact)

[#]Both authors contributed equally

[1] Department of Cardiac Development and Remodeling, Max Planck Institute for Heart and Lung Research, Bad Nauheim, Germany

[2] Department of Pharmacology, Max Planck Institute for Heart and Lung Research, Bad Nauheim, Germany

[3] Member of the German Center for Cardiovascular Research (DZHK), member of the German Center for Lung Research (DZL)

[4] College of Animal Science and Technology, Sichuan Agricultural University, Chengdu, 611130, China

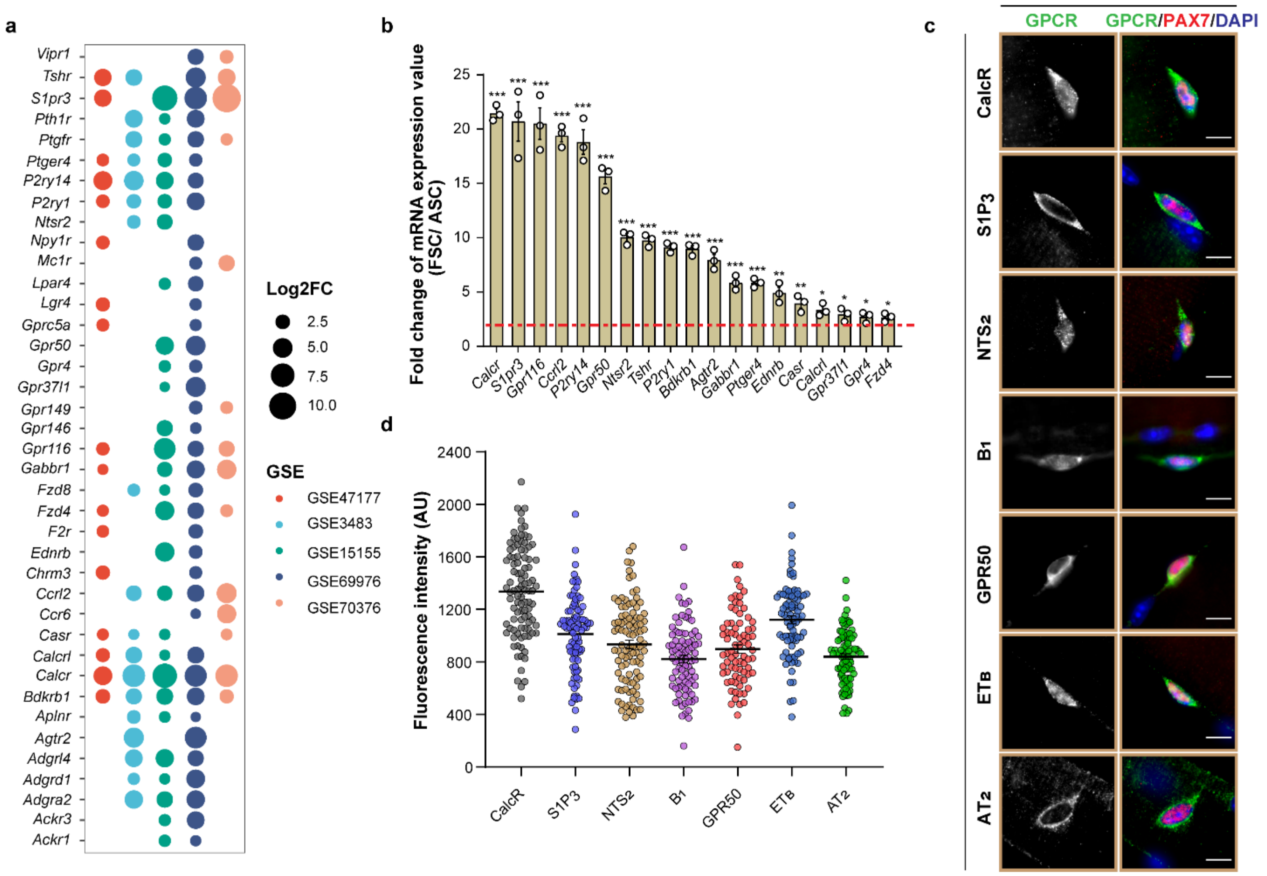

Supplementary Fig. S1: Identification of GPCRs highly expressed in quiescent MuSCs but downregulated upon activation.

a, Comparative analysis of gene expression in quiescent and activated MuSCs. The analysis was based on data extracted from 5 publicly available microarray datasets. Selected GPCRs show significant higher expression level (p<0.05, Log2_foldchange>1) in quiescent/freshly isolated MuSCs compared to G-alert/activated MuSCs in at least 2 different datasets. **b**, Taqman-assay based RT-qPCR expression analysis of selected *GPCRs* in freshly isolated MuSCs (FSC) and after 24h of culture (ASC). Expression levels were normalized to *Gapdh* (n=3). The red dashed line indicated a fold-change=2, unpaired t-test. **c**, **d**, Immunofluorescence for PAX7 (red), DAPI (blue), and GPCR (green) (**c**) and quantification (**d**) of GPCR staining intensity in MuSCs, situated on freshly isolated single FDB myofibers from WT mice (n=3). Scale bars in (**c**) represent 5 µm.

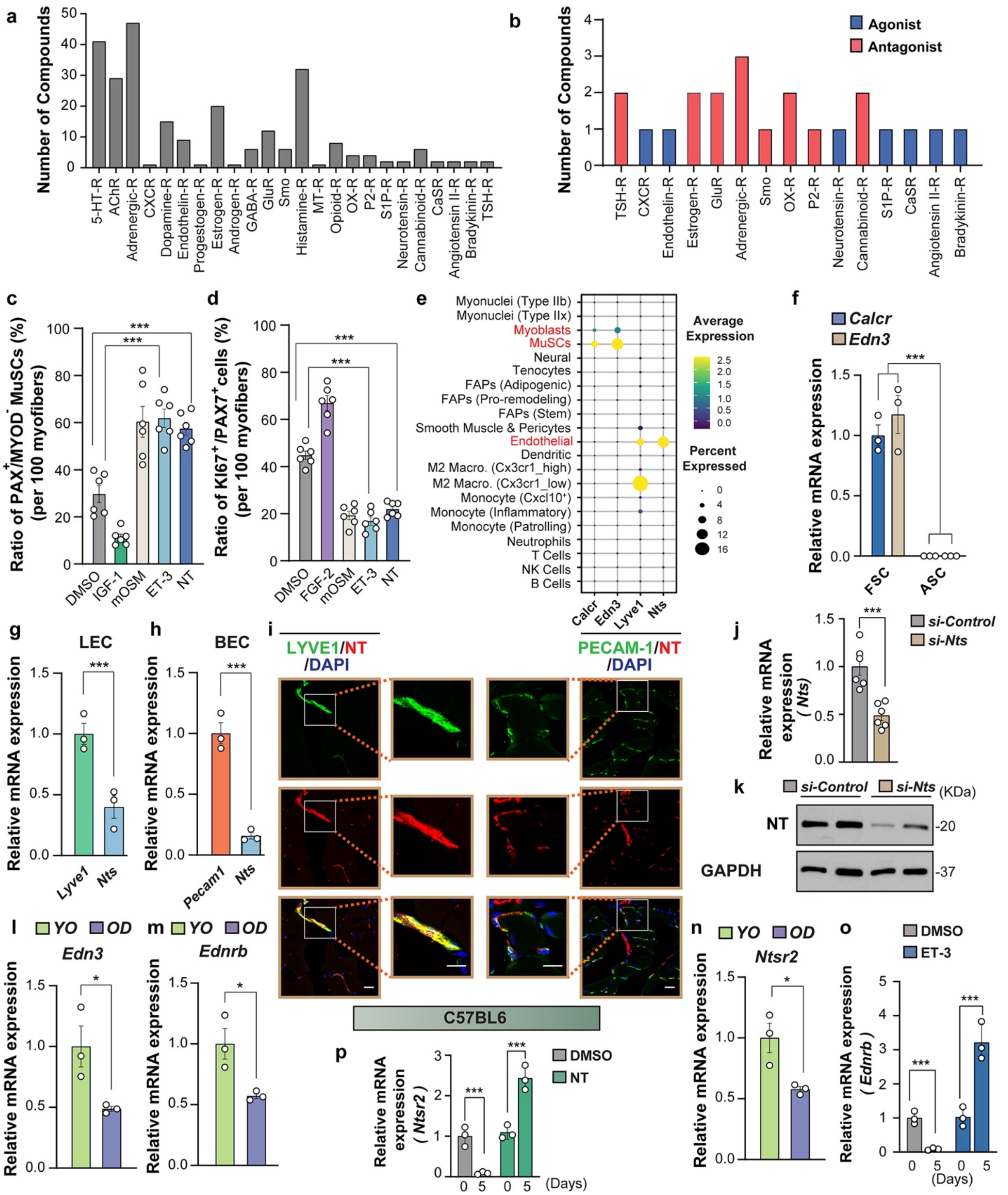

Supplementary Fig. S2: Origin of ET-3 and NT in mouse skeletal muscles

a, Composition of the customized GPCR compound library. **b**, Positively selected compounds and their targets, according to GPCR subfamilies. **c**, **d**, Ratios of PAX7^{+}MYOD^{-} (**c**) and PAX7^{+}KI67^{+} (**d**) MuSCs on isolated FDB myofibers after 24h exposure to DMSO, IGF1/FGF2, mOSM, ET-3,

and NT. (One-way ANOVA: *5 independent experiments, 100 myofibers per group*). **e**, Single-cell RNA-seq data from whole muscle of adult mice. Dot plots display expression of *Calcr, Edn3, Lyve1,* and *Nts* in different cell-type clusters. Dot size reflects the frequency of cells expressing non-zero transcript level. Dot colors indicate average expression levels. **f**, RT-qPCR analysis of *Calcr* and *Edn3* expression in freshly isolated MuSCs (FSC) and 24h of culture (ASC). Expression was normalized to *Gapdh* (One-way ANOVA: *n*=3). **g**, RT-qPCR analysis of *Lyve1* and *Nts* expression in lymphatic endothelial cells (LEC). Expression levels were normalized to *Gapdh* (unpaired t-test: *n*=3). **h**, RT-qPCR analysis of *Pecam1* and *Nts* expression in blood vessel endothelial cells (BEC). Expression levels were normalized to *Gapdh* (unpaired t-test: *n*=3). **i**, Representative images of immunostaining for NT (red), DAPI (blue), and LYVE1 or PECAM-1 (green) on TA muscle transverse sections of C57BL6 mouse. Scale bars represent 10 µm. **j**, RT-qPCR analysis of *Nts* mRNA levels in lymph endothelial cells (LECs) treated with siRNAs against *Nts* (*si-Nts*) compared to control siRNA (*si-Control*) 72 hours after transfection, normalized to *Gapdh* (unpaired t-test: *n*=3). **k**, Western blot analysis of NT protein levels under the same conditions as in (**j**). **l-n**, RT-qPCR analysis of *Edn3* (**l**), *Ednrb* (**m**)*,* and *Ntsr2* (**n**) expression in freshly isolated young (YO, 2 months old male C57BL6 mice) and old (OD, 24 months-old male C57BL6 mice) MuSCs. Expression levels were normalized to *Gapdh* (One-way ANOVA, *n*=3). **o**, **p**, RT-qPCR analysis of *Ednrb* (**o**) and *Ntsr2* (**p**) expression in MuSCs cultured in presence of DMSO, ET-3, and NT at the onset of the experiment (day 0) and after five days (day 5) of culture. Expression was normalized to *Gapdh* (One-way ANOVA, *n*=3).

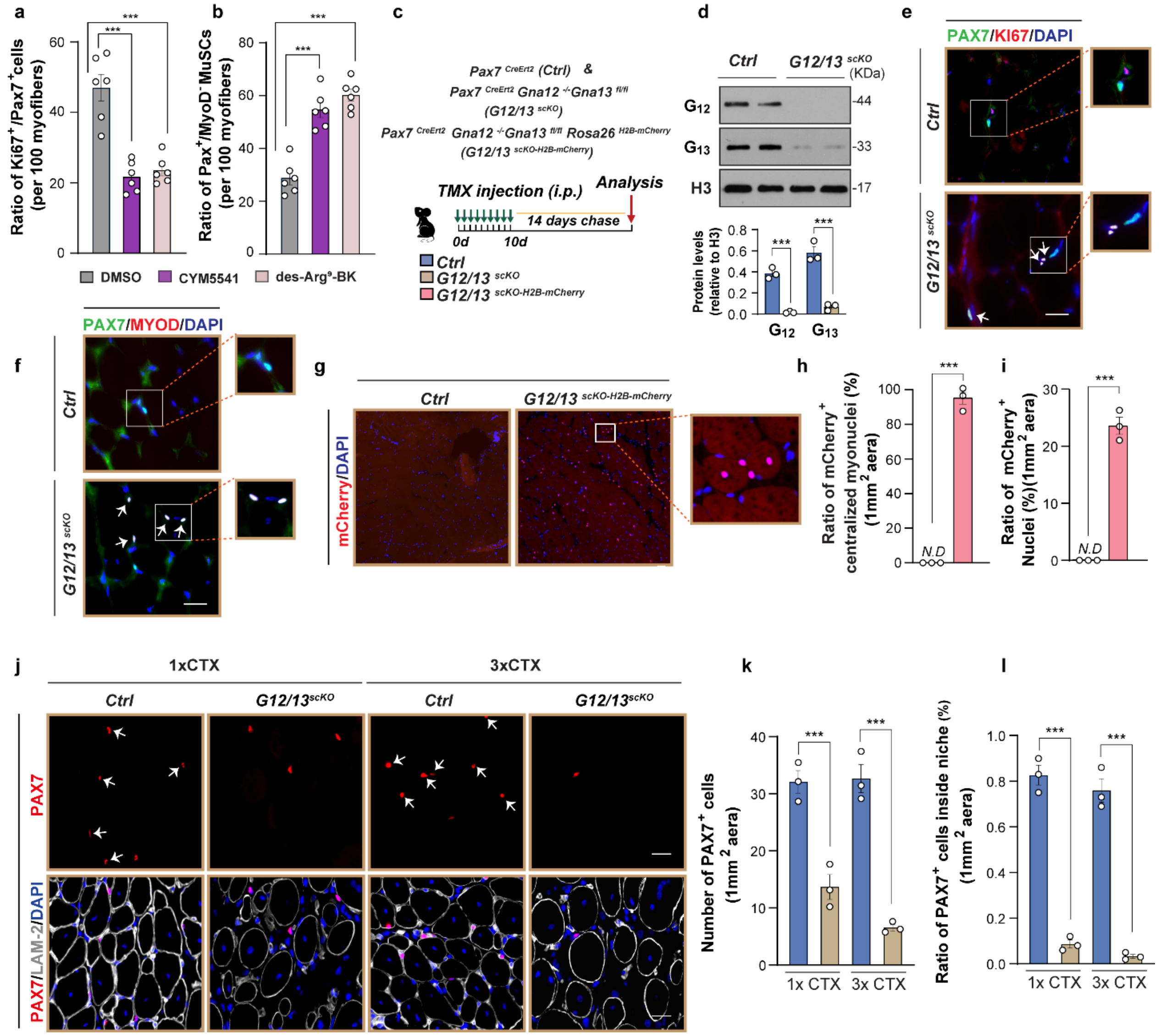

Supplementary Fig. S3: Inactivation of G_{12}-G_{13} abrogates MuSC quiescence and reduces the MuSC pool.

a, **b**, Ratios of $PAX7^{+}KI67^{+}$ (**a**) and $PAX7^{+}MYOD^{-}$ (**b**) MuSCs on isolated FDB myofibers after 24 h exposure to DMSO, CYM5541, and des-Arg^{9}-BK (des-Arg^{9}-Bradykinin). (One-way ANOVA: 6 independent experiments, 100 myofibers per group). **c**, Experimental design for (**d-i**). **d**, Western blots analysis of G_{12}, G_{13} and Histone3 (H3) expression in freshly isolated control and *G12/13*^{scKO} MuSCs. Quantification is in the lower panel. G_{12} and G_{13} levels were normalized to H3 (One-way ANOVA: $n = 3$). **e**, Immunofluorescence for PAX7 (green), KI67 (red), and DAPI (blue), in control and *G12/13*^{scKO} TA muscles. Scale bars represent 20 µm. **f**, Immunofluorescence for PAX7 (green), MYOD (red), and DAPI (blue), in control and *G12/13*^{scKO} TA muscles. Scale bars represent 20 µm. **g-i**, Immunofluorescence for mCherry (red) and DAPI (blue) in control and *G12/13*^{scKO} TA muscles

(**g**), ratios of centralized mCherry$^+$ myonuclei (**h**), and mCherry$^+$ nuclei in the periphery of myofibers (**i**) (unpaired t-test: n=3). Scale bars in (**g**) represent 20 μm. **j**, Immunofluorescence for PAX7 (red), LAMININ-2 (LAM-2) (grey), and DAPI (blue) in control and *G12/13*^{scKO} TA muscles 20 days post 1 time or 3 consecutive CTX-induced injuries. White arrows marke niche located PAX7$^+$ cells (One-way ANOVA: n = 3). Scale bars represent 10 μm. **k**, Quantification of PAX7$^+$ cells in control and *G12/13*^{scKO} TA muscles 20 days post 1 time or 3 consecutive CTX-induced injuries. **l**, The ratio of PAX7$^+$ cells in the niche to the total number of PAX7$^+$ cells in control and *G12/13*^{scKO} TA muscles 20 days post 1 time or 3 consecutive CTX-induced injuries (One-way ANOVA: n = 3).

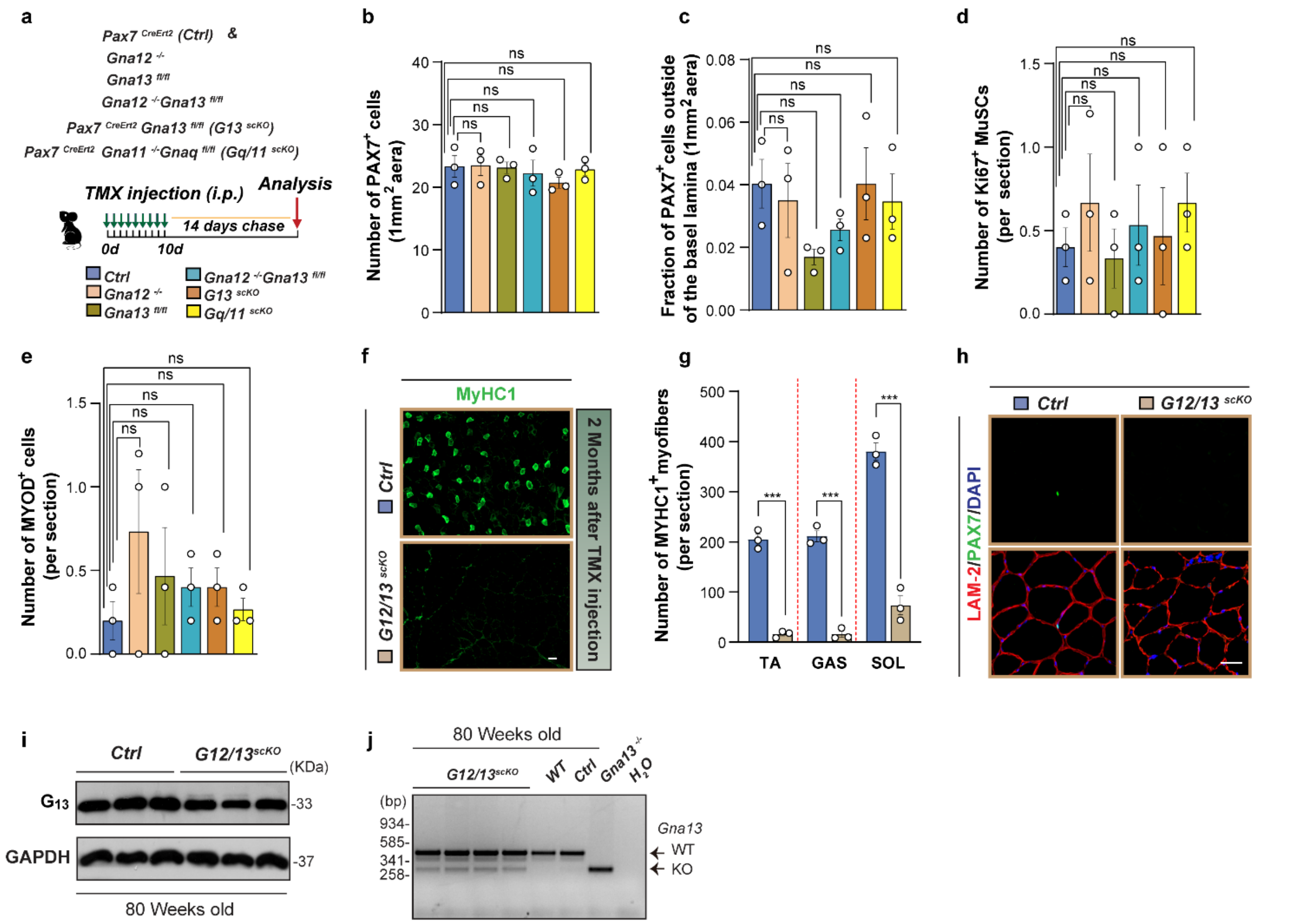

Supplementary Fig. S4: Prolonged absence of G_{12}-G_{13} in MuSCs is associated with increased muscle atrophy during aging.

a, Experimental design of (**b-e**). **b-e**, Quantifications of PAX7$^+$ cells (**b**), the fraction of MuSCs localized outside the basal lamina (**c**), KI67$^+$ MuSCs (**d**), and MYOD$^+$ cells (**e**) in TA muscles of control, *G12*$^{-/-}$, *Gna13*$^{fl/fl}$, *Gna12*$^{-/-}$ *Gna13*$^{fl/fl}$, *G13*^{scKO}, and *Gq/11*^{scKO} mice (One-way ANOVA: *n*=3, 5 sections per muscle sample). **f**, **g**, Immunofluorescence for MyHC1 (green), DAPI (blue) in control and *G12/13*^{scKO} TA muscles, 2 months after tamoxifen (TMX) injection (**f**), and quantification of MyHC1$^+$ myofibers in TA, GAS, and SOL muscles (**g**) (unpaired t-test: *n*=3, 17-weeks-old male mice, 5 sections per muscle sample). Scale bars in (**f**) represent 50 µm. **h**, Immunofluorescence staining for PAX7 (green), Laminin-2 (LAM-2) (red), and DAPI (blue) in aged control and *G12/13*^{scKO} TA muscles. Scale bars represent 10 µm. **i**, Western blot analysis of G_{13} protein levels in EDL myofibers from aged (80 weeks-old, male) control and *G12/13*^{scKO}. **j**, Genotyping of single EDL (extensor digitorum longus) myofibers isolated from 80-weeks-old *G12/13*^{scKO}, *Pax7* ^{CreErt2}(*Ctrl*), and C57BL6 (*WT*) mice. Each lane represents the DNA sample of

an individual myofiber. The lower band indicates the *Gna13* mutant allele. DNA extracted from a *Gna13$^{f/f}$ CMV-Cre* mouse embryo served as a positive control.

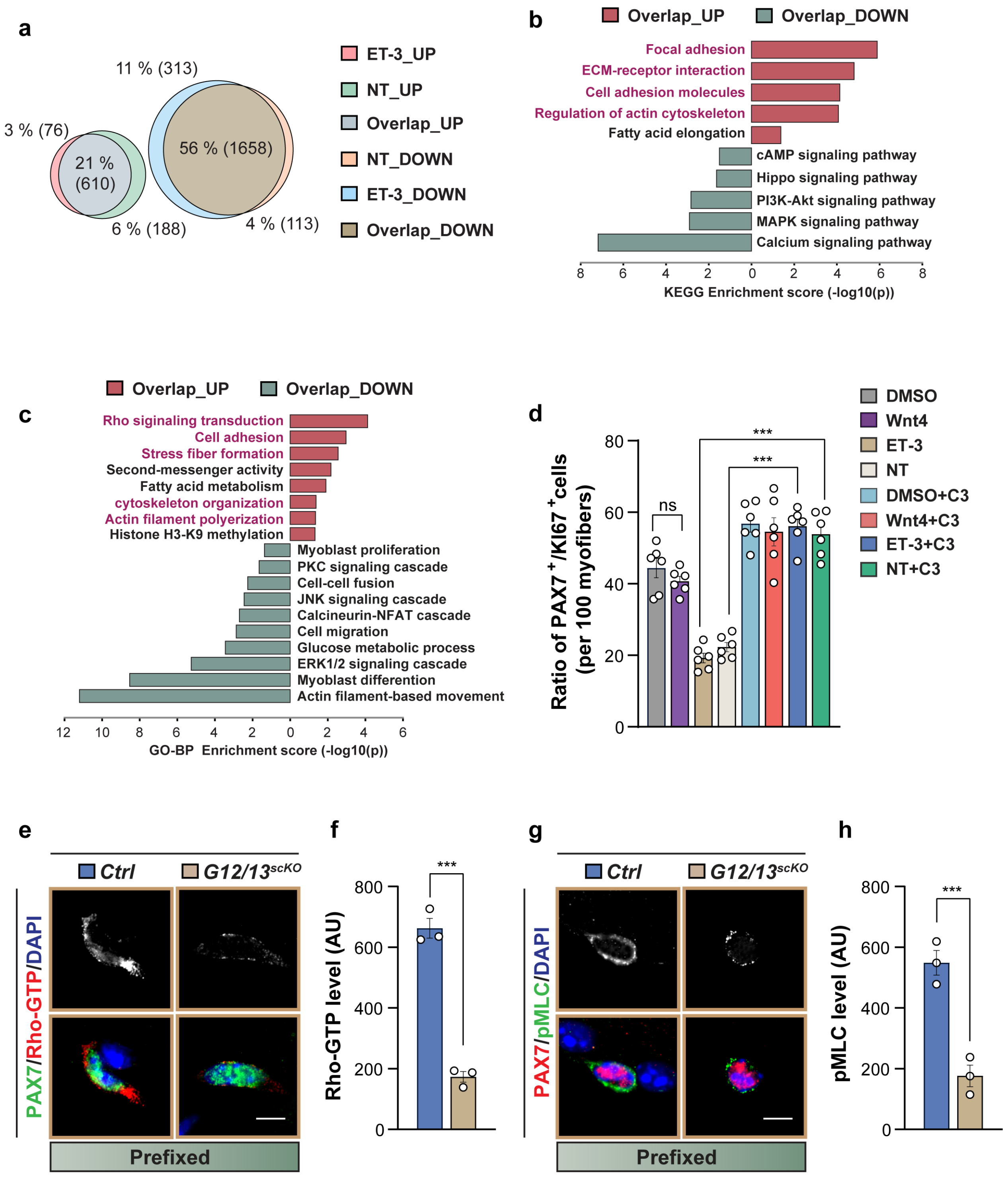

Supplementary Fig. S5: ET-3 and NT-induced G_{12}-G_{13} signaling relies on RhoA for maintaining quiescence of MuSCs.

a, Venn diagram, depicting the overlap of upregulated (left panel, genes with $p < 0.05$ and Log2foldchange > 1) and downregulated (right panel, genes with $p < 0.05$ and Log2foldchange < -

1) genes in ET-3 and NT compared to DMSO-treated MuSCs. **b**,**c**, KEGG (**b**) and Gene ontology (GO) analyses (**c**) of upregulated and downregulated genes, overlapping between ET-3 and NT-treated MuSCs compared to DMSO treatment. **d**, Ratios of $PAX7^{+}KI67^{+}$ MuSCs on isolated wild type FDB myofibers after 24h exposure to DMSO, ET-3, or NT, with or without Rho inhibitor (C3). (One-way ANOVA: *6 independent experiments, 100 myofibers per group*). **e**,**f**, Immunofluorescence for Rho-GTP (red), DAPI (blue), and PAX7 (green) (**e**) and quantification (**f**) of active Rho levels in MuSCs on isolated FDB myofibers of control and $G12/13^{scKO}$ mice (unpaired t-test: *n*=3). Scale bars in (**e**) represent 5 µm. **g**, **h**, Immunofluorescence for PAX7 (red), DAPI (blue), and pMLC (green) (**g**) and quantification (**h**) of pMLC levels in MuSCs on isolated FDB myofibers of control and $G12/13^{scKO}$ mice (unpaired t-test: *n*=3). Scale bars in (**g**) represent 5 µm.

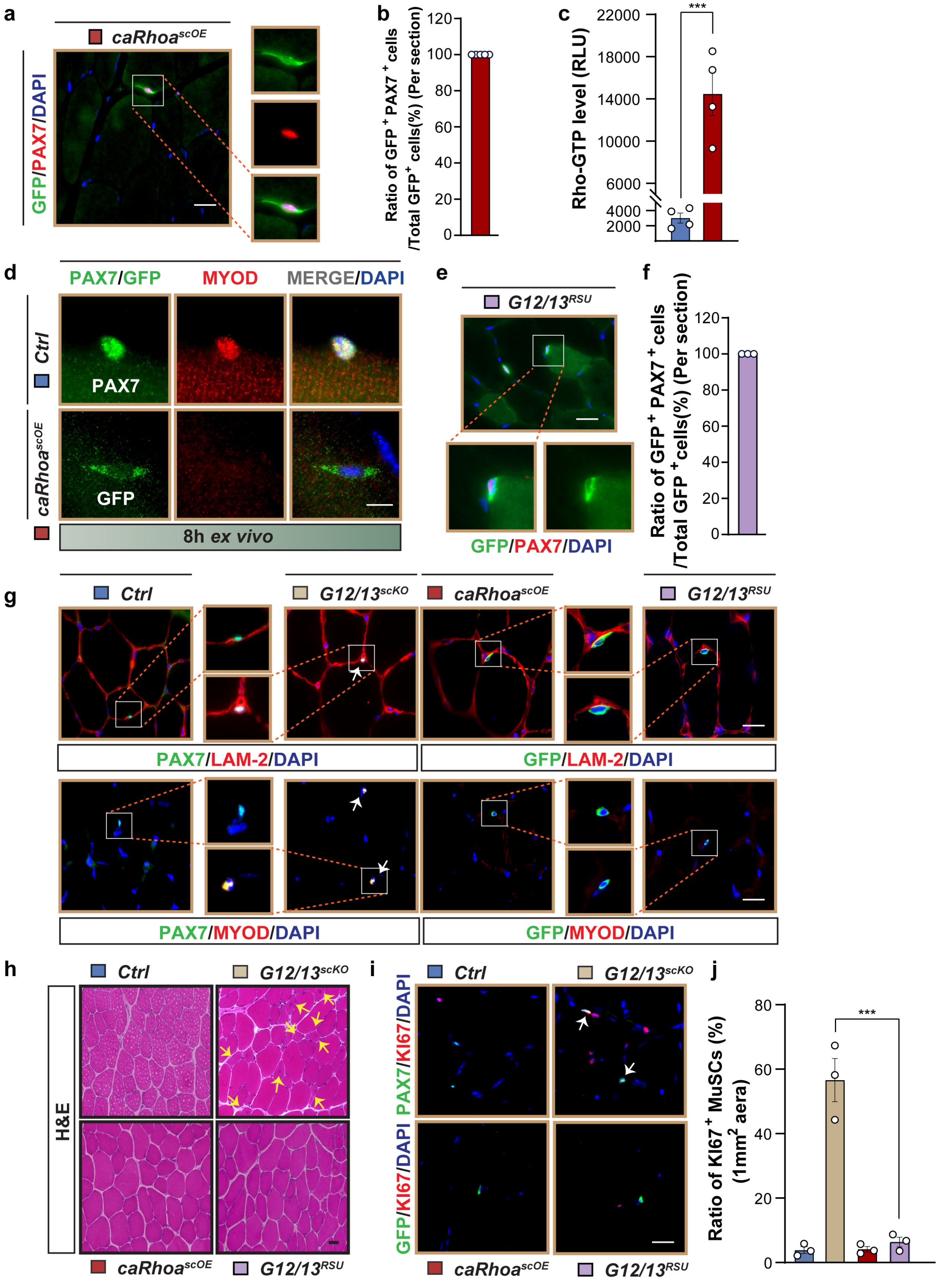

a
caRhoa^scOE
GFP/PAX7/DAPI
b
Ratio of GFP+ PAX7+ cells /Total GFP+ cells(%) (Per section)
c
Rho-GTP level (RLU)
***
d
PAX7/GFP
MYOD
MERGE/DAPI
Ctrl
caRhoa^scOE
PAX7
GFP
8h ex vivo
e
G12/13^RSU
GFP/PAX7/DAPI
f
Ratio of GFP+ PAX7+ cells /Total GFP+cells(%) (Per section)
g
Ctrl
G12/13^scKO
caRhoa^scOE
G12/13^RSU
PAX7/LAM-2/DAPI
GFP/LAM-2/DAPI
PAX7/MYOD/DAPI
GFP/MYOD/DAPI
h
H&E
i
PAX7/KI67/DAPI
GFP/KI67/DAPI
j
Ratio of KI67+ MuSCs (%) (1mm2 aera)
***

Supplementary Fig. S6: Constitutively active RhoA suppresses MuSC activation caused by inactivation of *Gna12-Gna13*.

a, **b**, Immunofluorescence for PAX7 (red), DAPI (blue), and GFP- caRhoA fluorescence (green) in $caRhoa^{scOE}$ TA muscles (**a**), and ratios of GFP $^{+}$Pax7^{+}cells in all GFP $^{+}$ cells (**b**) (*n*=5, 2-months-old male mice, 5 sections per muscle sample). Scale bars in (**a**) represent 10 µm. **c**, Rho activity measured by the G-LISA Kit in control and $caRhoa^{scOE}$ MuSCs after culture for 5 days. Active RhoA-GTP was determined by luminescence at 490 nm. (unpaired t-test: *n*=4, 3 replicates per group) **d**, Immunofluorescence for PAX7 (green), MYOD (red), DAPI (blue), and GFP-caRhoA (green) in MuSCs on isolated FDB myofibers of control and $caRhoa^{scOE}$ mice after 8h of culture. Scale bars represent 5 µm. **e**, **f**, Immunofluorescence for PAX7 (red), DAPI (blue) and GFP-caRhoA (green) in control and $G12/13^{RSU}$ TA muscles (**e**), and ratios of GFP $^{+}$Pax7^{+}cells in all GFP^{+} cells (**f**) (*n*=3, 5 sections per muscle sample). Scale bars in (**e**) represent 10 µm. **g**, Immunofluorescence for PAX7 (green), KI67 (red), DAPI (blue) and GFP-caRhoA (green) in control, $caRhoa^{scOE}$, $G12/13^{scKO}$, and $G12/13^{RSU}$ TA muscles. Scale bars represent 10 µm. **h**, H&E staining of control, $caRhoA^{scOE}$, $G12/13^{scKO}$, and $G12/13^{RSU}$ TA muscle sections. Scale bar represents 20 µm. **i**, **j**, Immunofluorescence for PAX7 (green), KI67 (red), DAPI (blue) and GFP-caRhoA (green) in control, $caRhoa^{scOE}$, $G12/13^{scKO}$, and $G12/13^{RSU}$ TA muscles (**i**), and ratios of KI67^{+}PAX7^{+} MuSCs in all PAX7^{+} MuSCs (**j**) (One-way ANOVA: *n*=3, 5 sections per muscle sample). Scale bars in (**j**) represent 10 µm.

Supplementary Table S1: List of reagent and resource used in this study. (Supplementary Table S1 in Suppl Tables.xlsx)

Supplementary Table S2: List of primers for genotyping or qRT-PCR. (Supplementary Table S2 in Suppl Tables.xlsx)

Supplementary Table S3: Compound list and information of the GPCR compounds library. (Supplementary Table S3 in Suppl Tables.xlsx)
